# AGL15 Promotion of Somatic Embryogenesis: Role and Molecular Mechanism

**DOI:** 10.3389/fpls.2022.861556

**Published:** 2022-03-28

**Authors:** Sanjay Joshi, Priyanka Paul, Jeanne M. Hartman, Sharyn E. Perry

**Affiliations:** ^1^Department of Plant and Soil Sciences, University of Kentucky, Lexington, KY, United States; ^2^Kentucky Tobacco Research and Development Center, University of Kentucky, Lexington, KY, United States

**Keywords:** somatic embryo, regeneration, MADS-box, transcription factor, chromatin immunoprecipitation, transcriptome, *Arabidopsis thaliana*

## Abstract

Plants have amazing regenerative properties with single somatic cells, or groups of cells able to give rise to fully formed plants. One means of regeneration is somatic embryogenesis, by which an embryonic structure is formed that “converts” into a plantlet. Somatic embryogenesis has been used as a model for zygotic processes that are buried within layers of maternal tissues. Understanding mechanisms of somatic embryo induction and development are important as a more accessible model for seed development. We rely on seed development not only for most of our caloric intake, but also as a delivery system for engineered crops to meet agricultural challenges. Regeneration of transformed cells is needed for this applied work as well as basic research to understand gene function. Here we focus on a MADS-domain transcription factor, AGAMOUS-Like15 (AGL15) that shows a positive correlation between accumulation levels and capacity for somatic embryogenesis. We relate AGL15 function to other transcription factors, hormones, and epigenetic modifiers involved in somatic embryo development.

## Introduction

Somatic embryogenesis (SE) is a powerful resource and biotechnological tool used for plant improvement, micropropagation, and clonal regeneration (Guan et al., 2016; Ochoa-Alejo and Loyola-Vargas, 2016). Because zygotic embryos (ZE) are embedded deep in maternal tissues, SE has become a widely used technique to understand processes that occur during zygotic embryogenesis. Somatic cells that undergo reprogramming to induce embryonic development produce structures called somatic embryos.

Understanding SE is important for several other basic and applied scientific approaches that are distinct from using SE as a model for ZE. Earth is in jeopardy due to climate change. Not only will crops suffer from abiotic stresses such as drought and heat, but also “new” biotic stresses that can dominate new territories due to the altered climate. While one hopes we can deal with this problem at the source, it seems increasingly likely we will need alternative strategies to deal with the evolving climate. One such strategy to continue to feed our growing population is biotechnology to engineer crops to cope with different environmental stressors and to propagate the most valuable genotypes as efficiently as possible (Ikeda and Kamada, 2005; Tian et al., 2020a). One challenge to genetic engineering is the introduction of the desired modifications into the genome. Another is the regeneration of a plant from the modified cell(s), and SE is one means to accomplish this latter task, although many plants and even particular genotypes do not regenerate well by SE. SE is of particular interest in plant developmental biology because of its involvement in plant plasticity, especially controlling the totipotency and pluripotency in somatic cells (Ikeuchi et al., 2016; Fehér, 2019). Interestingly, there are some parallels with animal cells acquiring the ability to dedifferentiate in response to stress (Grafi, 2009).

Several studies have been performed to decipher the regulatory mechanism of SE, and the model system *Arabidopsis thaliana* (Arabidopsis) has been used for much of this work (Yang and Zhang, 2010). Arabidopsis is a model plant due to its available genetic resources, with a small genome size and whole-genome sequence available from an early date (Kaul et al., 2000). These facts and its ability to be transformed very easily compared to other species that require intensive tissue culture (Clough and Bent, 1998) means that there is a database of mutants with T-DNAs inserted into nearly every gene-of-interest, generating a loss-of-function mutant that facilitates understanding gene function (https://conf.arabidopsis.org/display/COM/Mutant+and+Mapping+Resources). Arabidopsis zygotic embryogenesis has a predictable pattern and basic body plan from early division (Palovaara et al., 2016; Plotnikova et al., 2019), and SE generally follows the same stages. While there are differences in molecular profiles between ZE and SE, there are also congruencies worthy of further investigation (Tian et al., 2020a). Like ZE, SE generates bipolar structures from somatic cells with no vascular connection to the explant tissue (Thorpe and Stasolla, 2001; Von Arnold et al., 2002). A plethora of literature discusses genes encoding transcription factors (TF) that are involved in seed development and can promote SE or embryo-programs when ectopically expressed, including the so-called LAFL factors (*LEAFY COTYLEDON1 [LEC1], LEAFY COTYLEDON2* [*LEC2*], *FUSCA3 [FUS3]* and *ABSCISIC ACID INDPENDENT3 [ABI3]*) (Wójcikowska et al., 2013), *BABY BOOM* (*BBM*) (Boutilier et al., 2002; Jha and Kumar, 2018), *WUSCHEL* (*WUS*) (Jha et al., 2020), and *AGAMOUS-LIKE15* (*AGL15*) (Harding et al., 2003; Zheng et al., 2013a,b). Here we focus on AGL15 but include some discussion of relation to the other TFs as they interact in a complex network.

AGL15 is a member of the MADS domain TFs (named for **M**CM1 from *Saccharomyces cerevisiae*, **A**GAMOUS from Arabidopsis, **D**EFICIENS from *Antirrhinum majus*, **S**RF from *Homo sapiens*) (Gramzow and Theissen, 2010; Chen et al., 2017) that is expressed and primarily accumulates during early stages of seed development, and mostly within the embryo (Heck et al., 1995; Perry et al., 1996). The transcript level of *AGL15* in Brassica napus, and Arabidopsis, remains high during embryo morphogenesis until the seeds start to dry (Perry et al., 1996). Ectopic constitutive expression of *AGL15* promotes SE in Arabidopsis, *Glycine max* (soybean), and *Gossypium hirsutum* (cotton), while mutants (loss-of-function) of both AGL15 and AGL18 (closely related to AGL15) significantly reduce SE in Arabidopsis (Harding et al., 2003; Thakare et al., 2008; Zheng and Perry, 2014). Like *AGL15, AGL18* is able to promote SE when ectopically expressed in soybean and Arabidopsis (Zheng and Perry, 2014; Paul et al., 2021).

Here we focus on genes directly regulated by AGL15 to hypothesize about the stage of SE impacted by overexpression of *AGL15*. We highlight how AGL15 takes part in different processes involved in expression of genes to promote somatic embryogenesis.

## Stages of Somatic Embryogenesis (SE)

In SE, the process initiates when somatic cells are induced to change fate and form embryogenic cells that can eventually regenerate into a complete plant. This process can be direct or indirect (Sharp, 1980) with the latter including a callus phase. Many factors influence the ability to undergo SE, including plant type and even particular cultivars within a species, physiological status of the donor plant, tissue source and age of the explant, media components, and a variety of stresses (Yang and Zhang, 2010).

**Figure 1:**
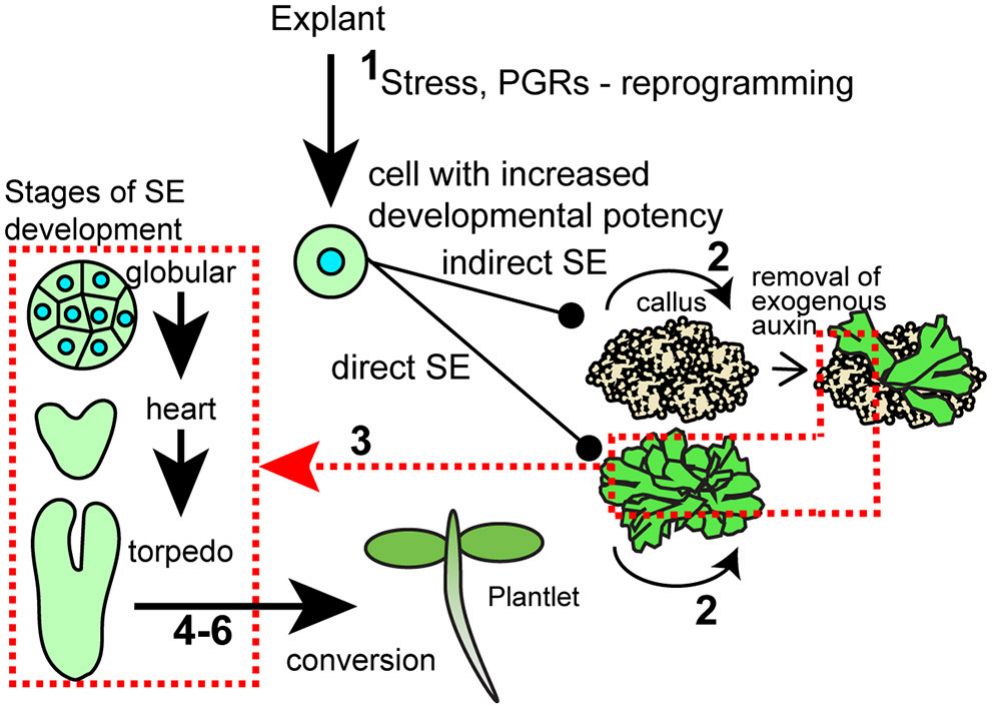
Diagram of stages of SE. Please see the text for a description of what the numbers mean.

Recent reviews have addressed the terms dedifferentiation vs. trans-differentiation. While dedifferentiation has been used to describe the switch from a somatic cell to an “undifferentiated” callus, it is increasingly recognized that there are no truly undifferentiated cells in plant regenerative processes. In fact, callus was recently recognized as having molecular profiles similar to root meristem tissue (Sugimoto et al., 2010). Dedifferentiation is still used to refer to a decrease in specialization, whereas trans-differentiation refers to a change from one cell type to a different cell type (Fehér, 2019; Bidabadi and Jain, 2020). While particulars to induce SE vary, a “theme” to induce SE is the treatment of explants with auxin, usually 2,4-D, a synthetic auxin that may act as an auxin, a stressing reagent, and/or to induce endogenous auxin production. Stress can also take the form of wounding, temperature, and nutritional stresses, among others. This process is thought to promote developmental switching via a series of molecular events and signal cascades resulting in the transition of somatic cells to embryo identity, or callus for indirect systems (Yang and Zhang, 2010; Fehér, 2015). The unique developmental process can be distinguished into two phases: the initiation/induction step and the developmental phase. Cell proliferation and increased plasticity of somatic cells occur in the first step, whereas cells differentiate to form somatic embryos when the culture cells get the right stimuli during the developmental phase (Magnani et al., 2017). A brief overview of different steps follows and is shown in Figure 1.

1. Initiation/induction—This is the step where the establishment of pre-embryogenic and embryogenic cells or groups of cells from the primary explant takes place. In this stage, plant growth hormones like auxin, and in some systems, cytokinin, play a vital role in generating cells responsive for the transition. Stresses are also commonly applied. Large epigenetic changes occur in response to these stimuli, and these can be seen at a cellular level by increased nuclear volume. The competent cells start activating the genes responsible for producing embryogenic cells (Su et al., 2021), including genes encoding TFs that then activate downstream pathways to generate the SE (Quiroz-Figueroa et al., 2006).
2. Proliferation/maintenance/multiplication—Once the cells are responsive and have entered embryogenetic fate, the proliferation of cells initiates, which can be bulked up usually on semisolid media or solid supports. Maintaining physical and chemical conditions, are crucial to maintaining the embryogenic process.
3. Maturation (histodifferentiation)—This involves morphological stages of somatic embryogenesis analogous to zygotic embryo development including establishment of shoot and root apical meristems. During this step, auxin is often removed from the media. For instance, often in indirect systems, SEs are only obvious upon removal or a decrease in the concentration of plant growth regulators (PGR). It is thought molecular patterns supporting development to the globular stage are established on the high PGR medium, and the decrease in exogenously added PGRs is needed to continue development (Zimmerman, 1993).
4. Post-maturation (drying, desiccation)—This stage involves mild drying that can switch the embryo from development to germination or for storage purposes.
5. *In vitro* germination (radicle elongation).
6. Conversion (establishment of the new plant).

## Systems Where Agl15 Has Been Found To Promote Somatic Embryogenesis (SE)

**Figure 2:**
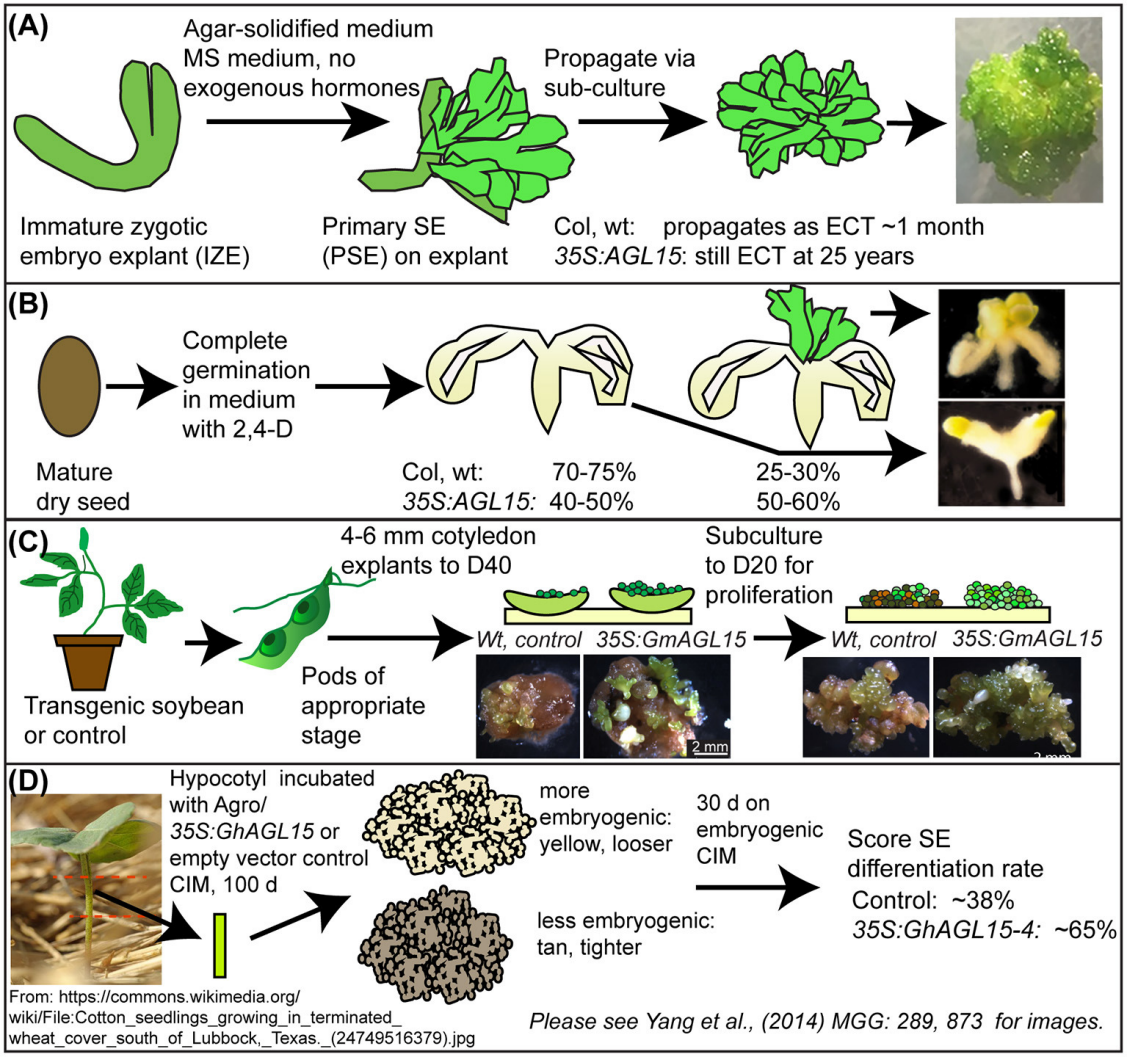
Systems where manipulation of AGL15 levels have impacted on SE. Modified from Tian et al. (2020a). **(A,B)** two systems in Arabidopsis; **(C)** in soybean; **(D)** in cotton. D40, medium with 40 mg/L 2,4-D, D20, medium with 20 mg/L 2,4-D, CIM, callus induction medium.

Ectopic overexpression of *AGL15* has been shown to promote SE in several plants (Figure 2). First, a system where Arabidopsis zygotic embryo explants are placed onto Murashige and Skoog (MS) medium lacking any exogenous plant growth regulators (PGR) show a positive correlation between AGL15 accumulation and ability to produce SE and maintain this development for long time periods (Harding et al., 2003). We refer to this tissue as Embryo Culture Tissue (ECT). Little to no apparent callus is observed in this system. While the Arabidopsis ecotypes Columbia (Col) and Wassilewskija (WS) wild-type (wt) embryos can transiently produce SE tissue in this system, they switch to leaves early. Meanwhile *35S:AGL15* increases production of ECT in WS and leads to long term maintenance for both ecotypes (25 years to date for WS; 10 years to date for Col) as SE tissue. Loss-of-function or dominant negative forms of AGL15 reduce initiation of this tissue. Because *AGL15* transcript accumulation is induced in response to auxin treatment (Zhu and Perry, 2005), one possibility is the overexpression of transgene removes the need for exogenous auxin. Kurczynska et al. (2007) demonstrated that the cells competent for SE are in the protodermis and subprotodermis of the adaxial side of the cotyledons. Whether the *35S:AGL15* transgene enhances this competence or expands it to other tissues remains a question for the future.

Apart from ECT, there has been another SE system where the mature seeds are allowed to complete germination in liquid media where synthetic auxin 2,4-D is present. After 21 days of culture, development at the shoot apex region of the seedlings is counted for those with SE tissue and those lacking this tissue. Other than SE development at the meristem, the remainder of the “seedling” is callused. We refer to this system as **S**hoot **A**pical **M**eristem **S**omatic **E**mbryogenesis (SAM SE). The findings show that *35S:AGL15* produced approximately twice the frequency of seedlings with SAM SE compared to Col, wt (for example, 56% for 35S:AGL15, compared to 29% for Col, wt; Zheng et al., 2009). In SAM SE, 2,4-D is required to obtain SE development; medium lacking 2,4-D simply produces liquid grown seedlings. However, different sensitivity to 2,4-D was observed for *35S:AGL15* compared to Col, wt (Zheng et al., 2016).

We expanded this work to *Glycine max* (soybean). Genes encoding orthologs of AGL15 from soybean (GmAGL15) and the closely related MADS-factor that has redundant functions (GmAGL18) were cloned and introduced into SE tissue by biolistic transformation. We found that increased expression of these genes via a *35S* promoter could 1. Enhance recovery of transgenic lines, presumably by promoting regeneration of transformed cells by SE and 2. Produce transgenic plants that have enhanced ability to form SE tissue. Meanwhile, Yang et al. (2014) cloned three AGL15 orthologs from cotton and demonstrated that overexpression could promote SE in this plant.

**Figure 3:**
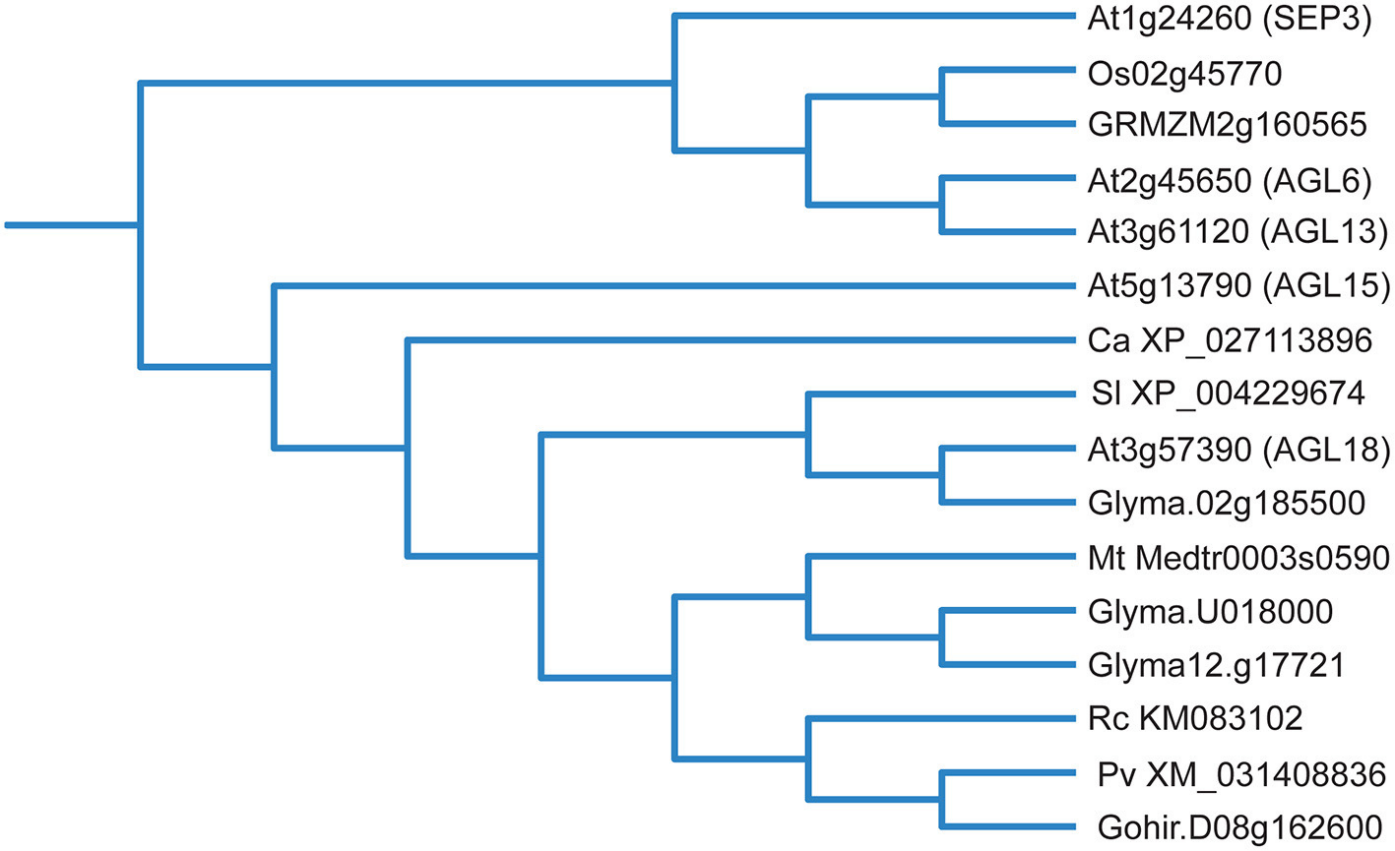
Phylogenetic tree of selected potential AGL15 orthologs, some of which promote SE development (please see text). Protein sequences were retrieved from Phytozome (Goodstein et al., 2012) and submitted to the phylogenetic analysis tool NGPhylogeny.fr (Lemoine et al., 2019) with default settings. The output tree was then uploaded to iTOL (version 6.5) (Letunic and Bork, 2019) for tree visualization. *At, Arabidopsis thaliana; Os, Oryza sativa; GRMZM, Zea mays; Ca, Coffea arabica; Sl, Solanum lycopersicum; Glyma, Glycine max; Mt, Medicago truncatula; Rc, Rosa canina; Pv, Pistacia vera; Gohir, Gossypium hirsutum*.

While this summarizes the studies where AGL15 accumulation has been manipulated to show effects on SE development, other studies show a correlation between SE and AGL15 accumulation. Potential orthologs of AGL15 are shown in Figure 3. Perry et al. (1999) demonstrated that embryos from different sources, including apomictic, microspore embryos, and SE accumulated protein that reacted with AGL15 antiserum. Tvorogova et al. (2019) overexpressed another TF called *MtWOX9* in *Medicago truncatula* and found increased SE capacity. *MtAGL15* transcript accumulation increased in response to MtWOX9. In addition, a highly embryogenic line called 2HA showed increased *MtAGL15* transcript with SE formation. The non-embryogenic line (A17) showed no such increase. Ghadirzadeh-Khorzoghi et al. (2019) showed increased *PvAGL15* transcript in embryogenic explants of pistachio, while Xu et al. (2018) showed *RcAGL15* associated with protocorm-like bodies in rose.

## Gene Regulation By Agl15 During Somatic Embryogenesis

AGL15 is a central regulator in SE and to understand how it directly and indirectly controls this process, expressed and repressed target genes were identified using chromatin immunoprecipitation (ChIP) and transcriptome studies in Arabidopsis and soybean (Zheng et al., 2009; Zheng and Perry, 2014). Direct targets (Arabidopsis where a genome-wide study was performed) were found to be enriched for a *cis* motif called a CArG motif that is a binding site for MADS-factors like AGL15. The consensus CArG is CC(A/T)6GG, but AGL15 can also bind variants of this motif with a longer A/T rich core (Tang and Perry, 2003). Studies to determine how ectopic expression of TFs promote SE have resulted in strategies to enhance SE from non-transgenic tissue. Examples for AGL15 include manipulation of gibberellin metabolism and ethylene biosynthesis and response (discussed further below).

**Figure 4:**
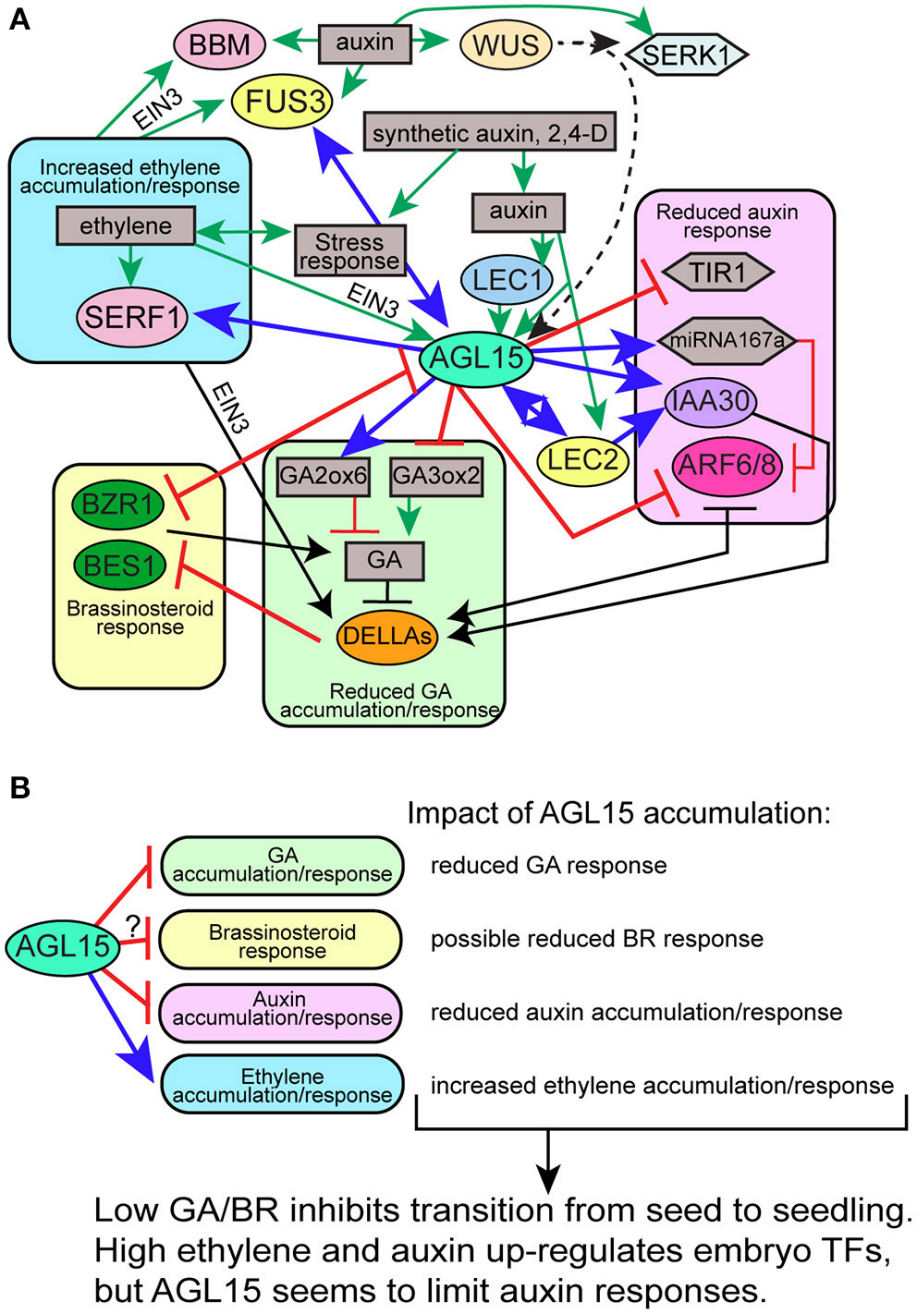
Select interactions between TFs involved in SE and control of hormone accumulation/signaling. Not all interactions are shown in consideration of clarity. **(A)** Particular gene interactions; **(B)** summary of tested or presumed AGL15 effects on different hormones. Different colored ovals with TF names indicate different families of TFs: yellow, B3 domain; blue, CCAAT-box binding; pale pink, APETALA2 (AP2) or AP2/ERF; mint, MADS; dark green, BZR domain; lavender, AUX/IAA domain; orange, DELLA/GRAS; light orange, homeodomain. Gray boxes indicate hormone or other responses. Gray hexagon, miRNA; light blue hexagon, SERK1.

AGL15 interacts in a complex network with several other TFs that promote SE, including members of the LAFL TFs, BBM, and WUS. This was recently reviewed in Tian et al. (2020a), and some of these interactions are highlighted in Figure 4.

Comparison of targets of AGL15 in Arabidopsis and soybean revealed some intriguing similarities. In both species, genes involved in stress responses were overrepresented among the direct responsive targets. These include a set of WRKY, NAC, and bZIP TFs that have been associated with dedifferentiation (Grafi et al., 2011). A more recent ChIP-seq study for Arabidopsis AGL15 revealed that regulatory regions corresponding to genes encoding nearly all of these TFs are directly bound by AGL15, which seems quite extraordinary (Paul et al., 2021). When comparing the percentage of genes encoding these TFs bound by AGL15 to the percentage of genes bound overall compared to the whole genome, a significant enrichment in the dedifferentiation-related (DD) TFs potentially directly regulated by AGL15 is observed (Table 1). Interestingly, many of the other TFs that can promote SE also show significant enrichment of these genes among their direct targets (Table 1). Detailed information on what SE TF associates with what DD TF gene is provided in Supplementary Table 1.

**Table 1:** Overrepresentation of TFs associated with dedifferentiation (DD TFs) among the genes with regions bound by TFs involved in SE.

**TF**	**# DD TFs bound**	**Total # of genes bound**	**Significance**	**References**
LEC1, early SE	13	3,035	0.00010	(Pelletier et al., 2017)
LEC1, late SE	17	4,890	0.00035	(Pelletier et al., 2017)
LEC1, cotyledon	22	7,216	0.00037	(Pelletier et al., 2017)
LEC2	19	2,788	5.2E-13	(Wang et al., 2020)
FUS3	4	1,218	NS	(Wang and Perry, 2013)
ABI3	14	2,510	1.5E-07	(Tian et al., 2020b)
BBM	12	Top 5,000	NS	(Horstman et al., 2015)
AGL15, ChIP-chip	15	3,708	7.6E-05	(Zheng et al., 2009)
AGL15, ChIP-seq	29	9,729	2.8E-05	(Paul et al., 2021)
AGL18, ChIP-seq	4	3,446	NS	(Paul et al., 2021)

*Chi-square test was used to compare the number of TFs associated with dedifferentiation (DD TFs; 44 total as listed in Grafi et al., 2011) bound by select regulators (TF) of embryogenesis, compared to the total list of genes bound for each TF, using 27,334 genes total for Arabidopsis (NCBI, accessed 11/17/2021). Significance means that within the list bound by the TF, DD TFs are overrepresented compared to expectations based on the total # of genes bound*.

**Figure 5:**
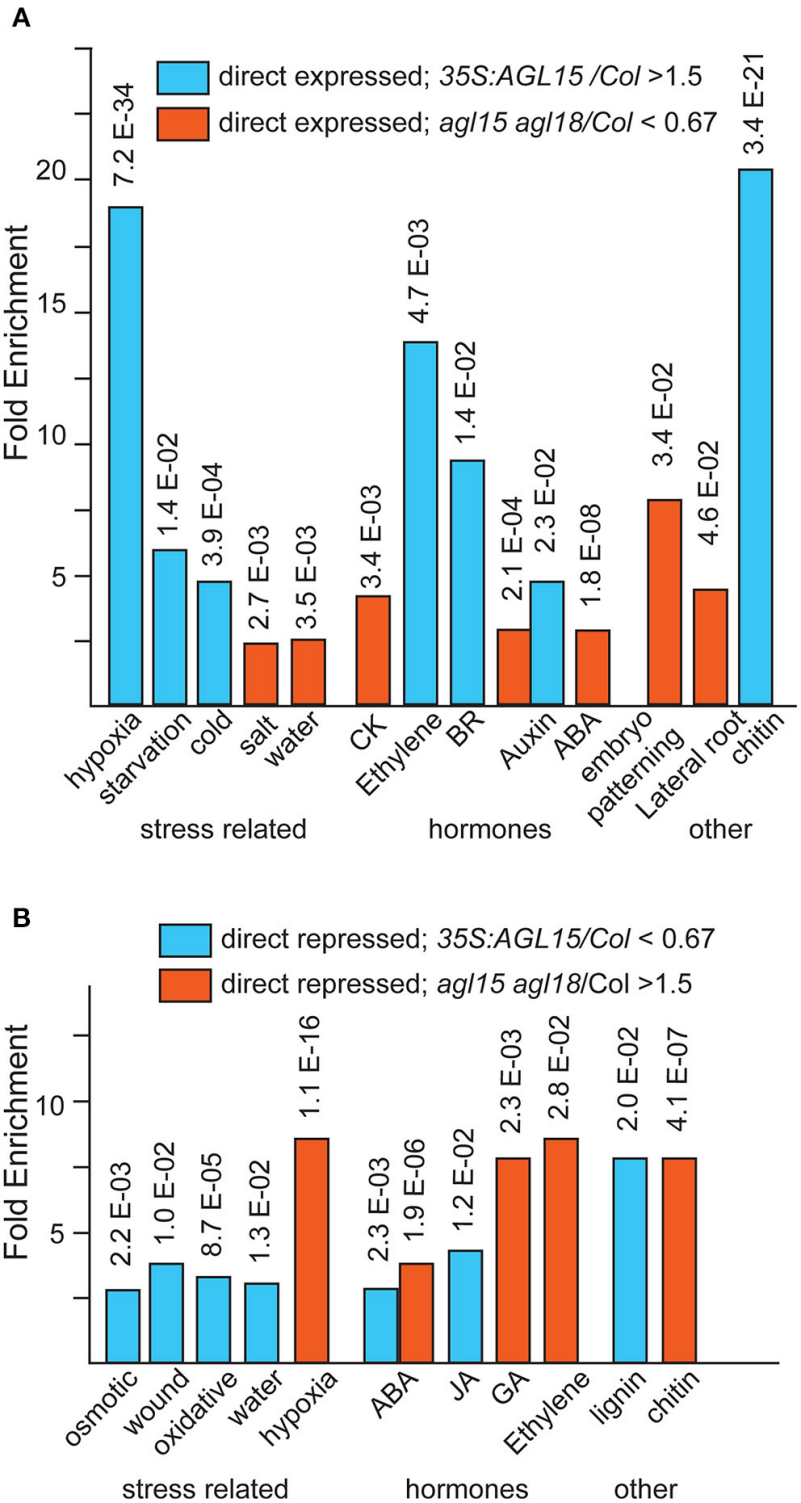
Overrepresented GO annotations among the direct (as measured in ChIP-chip and/or ChIP-seq) and responsive (at least a 1.5-fold change at *P* < 0.05). Data is from the Panther Classification system accessed July 9, 2021. **(A)** Directly expressed. **(B)** Directly repressed.

A number of gene ontology (GO) categories are overrepresented among the direct (as determined by ChIP-chip and/or ChIP-seq) AGL15 targets that are expressed or repressed. Select categories are shown in Figure 5. Notably, genes involved in abiotic stress response are overrepresented, as are hormone response, particularly hormones involved in stress. Stresses such as those shown in Figure 5 are often used to induce SE. Interestingly, genes associated with “chitin” are overrepresented among AGL15 direct expressed targets (Figure 5A). Several studies have found that lipophilic chitin oligosaccharides, endochitinases, and arabinogalactan proteins (AGP) have roles in stimulating SE (Van Hengel et al., 1998, 2002; Domon et al., 2000) and also are responsive to stress (reviewed in Grover, 2012; Mareri et al., 2019). One well-studied SE-related chitinase is EXTRACELLULAR PROTEIN3 (EP3) from *Daucus carota* (carrot), where addition of EP3 can rescue the production of SE in a SE-defective line of carrot (Van Hengel et al., 1998). In soybean, *35S:GmAGL15* was found to initially increase transcript accumulation from a gene encoding a potential EP3 ortholog in the explants. However, at later time points after culture on D40 medium, differential transcript accumulation compared to control was lost due to more extensive up-regulation in the control compared to the overexpressor (Zheng and Perry, 2014). *DcEP3* is not expressed in the somatic (or zygotic) embryos, but rather in cells surrounding the embryos and are thought to have a nursing function, in part by cleavage of AGPs, some of which have stimulatory and others inhibitory effects on SE (Toonen et al., 1997; Van Hengel et al., 2001). Some AGPs have been found to be important in the conditioned medium to promote SE, and at least some are expressed in the non-embryogenic cells in embryogenic cultures (reviewed in Von Arnold et al., 2002). Interestingly, AGPs, including the subgroup of fasciclin-like AGPs, (FLAs) were prevalent on the AGL15 direct repressed list (Zheng et al., 2009), a list that became longer with the ChIP-seq data (Paul et al., 2021) (Table 2). Only one (*AGP14*) showed direct expression in response to AGL15 accumulation, with one additional (*FLA10*) showing reduced transcript in both comparisons.

**Table 2:** *AGP* and *FLA* genes that show transcript response to AGL15 and are direct targets as determined by ChIP-chip or ChIP-seq.

**AGI**	**TAIR10**	**ChIP-chip**	**ChIP-seq**	***agl15agl18/Col***		***35S:AGL15/Col***	
				**fold-change**	***P*-value**	**fold-change**	***P*-value**
At1g03870	FLA9	Y	Y	2.26	0.03	0.41	0.16
At1g35230	AGP5	Y	Y	2.60	0.00	1.01	0.96
At2g04780	FLA7		Y	2.12	0.01	0.89	0.58
At2g14890	AGP9	Y	Y	1.82	0.01	0.81	0.50
At2g23130	AGP17	Y	Y	2.62	0.01	0.50	0.26
At2g45470	FLA8	Y	Y	2.56	0.01	0.57	0.29
At2g46330	AGP16		Y	1.79	0.00	1.13	0.26
At3g06360	AGP27		Y	0.82	0.28	0.65	0.05
At3g52370	FLA15	Y	Y	0.46	0.00	0.49	0.01
At3g60900	FLA10	Y		2.20	0.00	0.76	0.44
At4g09030	AGP10		Y	1.98	0.01	0.75	0.42
At4g12730	FLA2		Y	2.05	0.02	0.39	0.10
At4g37450	AGP18	Y	Y	1.66	0.02	0.41	0.06
At5g11740	AGP15	Y	Y	1.60	0.01	1.06	0.73
At5g56540	AGP14		Y	0.55	0.00	1.28	0.13
At5g64310	AGP1	Y		2.06	0.00	1.15	0.68

*Chip-chip is from Zheng et al. (2009), but allowing transcript change of 0.67–1.5. ChIP-seq is from Paul et al. (2021), analyzed by CLC Genomics Workbench, but also including the three sets of data analyzed by CisGenome (Ji et al., 2008). The transcriptomic data is from Zheng et al. (2009)*.

## Agl15 and Hormone Relations In Somatic Embryogenesis

Hormones/PGRs play a crucial role in SE induction. Thus, to understand SE, it is important to understand the signaling processes related to both the exogenous and endogenous PGRs. Generally, SE depends on the type and concentration of PGR used for each culture medium. Recent studies showed that different plant species, including Arabidopsis (Wójcikowska et al., 2013)*, Coffea canephora* (Márquez-López et al., 2018), and *Musa spp*. (Awasthi et al., 2017), successfully responded to SE induction using variable explants, conditions, and concentrations of PGRs. Although hormones are only part of the composition of a culture medium, it has been estimated that design of a new tissue culture medium requires optimizing components that can amount to 16,000 different treatments, leading to artificial intelligence/optimization algorithms to assist development (Hesami et al., 2020). Thus, understanding mechanisms of SE, including how TFs that can promote SE regulate and respond to PGRs, is important to help guide these efforts. Here, we focus on the relation between AGL15, select other TFs that promote SE and select PGRs, and the underlying mechanisms that regulate different hormone signaling pathways. A working model is shown in Figure 4. It should be noted that hormone interactions are exceedingly complex with cooperative or antagonistic interactions depending on the particular context, so this model is a simplification focusing on downstream targets of AGL15 and how they may interact to promote SE.

### AGL15 and Auxin

One of the most common components in culture media for SE is auxin, often supplied as 2,4-D, a synthetic auxin. As mentioned above, it has been proposed that this exogenously added PGR may act as an auxin, as a stressing agent, and/or to induce endogenous auxin production. Several TFs that can induce SE respond either directly or indirectly to auxin including BBM, FUS3, LEC1, LEC2, WUS, and AGL15 (reviewed in Tian et al., 2020a). While it does not encode a TF *per se, SOMATIC EMBRYOGENESIS RECEPTOR LIKE KINASE 1* (*SERK1*), is a key marker for cells able to form somatic embryos, and, when expressed via a strong constitutive promoter, can enhance SE production in multiple species. *SERK1* transcript accumulation is increased by auxin and stress (for a review, please see Mendez-Hernandez et al., 2019). Interestingly, SERK1 has been found to interact with protein complexes that include AGL15, as well as proteins involved in brassinosteroid signaling (Karlova et al., 2006 and please see below). Both *SERK1* and *AGL15* are positively (possibly indirectly) regulated by WUS (Busch et al., 2010). Further studies in Arabidopsis have demonstrated the importance of auxin in SE (Gaj et al., 2006) as well as an optimal 2,4-D treatment that can be explained, at least in part, by auxin effect on SE-related TF genes. All of the genes in the study showed increased and relatively stable (over the time course) transcript accumulation at the optimal 2,4-D concentration for SE (5 μM) (Grzybkowska et al., 2020). However, at 3 days of culture on various media, *LEC1, LEC2* (two of the previously mentioned LAFL factors), and *BBM* showed a positive correlation of transcript accumulation over the 2,4-D concentrations used, including sub-optimal (0.1 μM) that led to shoot development, 5 μM (optimal for SE), and supra-optimal (20 μM) that produced non-embryogenic callus, whereas *AGL15* and *WUSCHEL* (*WUS*) had very little or no transcript accumulation other than at the SE optimal 2,4-D concentration. Grzybkowska et al. (2020) further linked this response to epigenetic regulation (discussed below). While AGL15 is responsive to auxin (both 2,4-D and the biologically relevant auxin indole acetic acid, IAA), it is not an auxin early responsive gene and requires one to two-day treatment to see a clear response (Zhu and Perry, 2005). Consistent with this is the lack of an auxin response element in the promoter region; but auxin may act via an ethylene responsive element (Grzybkowska et al., 2020). Putative orthologs of AGL15 from cotton (Figure 3) are also significantly induced by 2,4-D, and when ectopically expressed, enhance the embryogenic potential of calli in 2,4-D containing medium (Yang et al., 2014). While it is unclear that monocots have MADS-factors that, when compared to Arabidopsis MADS, are most similar to AGL15, in *Zea mays AGL15*-like gene expression was correlated with early embryogenic culture initiation. For two of the four genes encoding these maize AGL15-like genes, transcript accumulation increased after placement on 2,4-D containing medium (Salvo et al., 2014). One of these genes is shown in Figure 3. However, the soybean orthologs of AGL15, (Glyma12g17721 and Glyma11g16105) did not respond to 2,4-D, although an AGL18 ortholog (GmAGL18) was upregulated in response to 2,4-D (Zheng and Perry, 2014).

The role of auxin in SE induction is of particular interest. Auxin treatment is important for embryogenesis (both somatic and zygotic); however, too high auxin concentration may hinder the induction of embryogenic cells in the SE culture. It has been observed that cells that are resistant to auxin in that they maintain cell-cell adherence and do not elongate as much, may be the particular cells capable of developing as embryos (Emons, 1994). In the Arabidopsis auxin signal transduction pathway, the *AUXIN/INDOLE-3-ACETIC ACID* (*AUX/IAAs*) encoded proteins bind to the Auxin Responsive Factors (ARFs), which, in turn, are associated with DNA via auxin response elements on auxin-responsive genes (Piya et al., 2014). AUX/IAAs act as repressors of ARF function by keeping ARFs from regulating gene expression until auxin perception (involving auxin receptors TRANSPORT INHIBITOR RESPONSE1; TIR1, and AUXIN SIGNALING F-BOX; ABFs) leads to degradation of the IAA protein.

While auxin is important for SE, and other TFs that can induce SE directly upregulate auxin biosynthetic genes (e.g., members of the *YUCCA* gene family are induced by LEC1 and LEC2), overexpression of *YUCCA*'s alone cannot induce SE (reviewed in Braybrook and Harada, 2008). It has been proposed that other factors, including IAA30 (discussed below) and/or AGL15, may be important for competency to respond to auxin. Part of this “competency” could be limiting auxin response.

AGL15 controls several genes in a manner that would reduce auxin response and/or accumulation. In soybean, we found that *35S:GmAGL15* reduces endogenous IAA accumulation compared to the wild type control in developing embryos (Zheng et al., 2016). In Arabidopsis, 35S:*AGL15* directly upregulated the gene encoding an AUX/IAA transcriptional repressor, IAA30, which may limit auxin responses and is important for SE (Zheng et al., 2009). An *iaa30* homozygous knockout line in both Col, wt and *35S:AGL15* backgrounds showed a significantly lower frequency of SAM SE development than in the corresponding background with the wild type *IAA30* gene (Zheng et al., 2009). *IAA30* is also a direct expressed target of LEC2 (Braybrook and Harada, 2008). Aux/IAAs generally contains four domains, including an N-terminal repression domain (domain I), domain II that enables degradation of Aux/IAAs via ubiquitin–proteasome pathway in response to auxin, and domains III and IV are the protein-protein interaction domains (Guilfoyle and Hagen, 2012). However, in Arabidopsis, IAA30 lacks the domain II that is involved in binding auxin F-box receptors in response to auxin perception. In addition, IAA20, which is also a potentially upregulated target of AGL15, also lacks domain II. As a result, unlike the canonical IAA proteins, these Arabidopsis IAA proteins have a longer half-life (Sato and Yamamoto, 2008) and overexpression of *IAA30* may interrupt the auxin responsiveness.

In addition (Gm)AGL15 (here (Gm)AGL15 is meant to indicate similar observations for soybean and Arabidopsis AGL15; AGL15 is meant to indicate the observation in Arabidopsis to date) directly represses a gene encoding an auxin receptor *(Gm)TIR1* (Zheng et al., 2016) that facilitates the degradation of AUX/IAA in response to auxin (Hayashi, 2012), and represses both *(Gm)ARF6* and *(Gm)ARF8* (Zheng et al., 2016), which are reported as transcriptional activators in the auxin signaling pathway (Piya et al., 2014). This regulatory interaction appears to be direct for *ARF6* in Arabidopsis with binding by AGL15 observed in ChIP experiments (Zheng et al., 2016). Both *ARF6* and *ARF8* are post-transcriptionally controlled by miRNA167 and AGL15 upregulates one of the genes encoding this microRNA, with evidence for a direct interaction in Arabidopsis (Zheng et al., 2016). Loss-of-function mutants of *arf6* and the double *arf6/arf8* showed a significant increase in SAM SE (Zheng et al., 2016), but in other SE systems (culture of immature zygotic embryos), there was a reduction in SE efficiency or productivity for the *arf6* and *arf8* single mutants (Wójcikowska and Gaj, 2017). Thus, context seems important.

It is important to mention here that AUX/IAAS, ARFs, and auxin receptors are multigene family proteins. Therefore, it is feasible that other (Gm)AGL15 regulated members may promote auxin response via other family members. For instance, there is data supportive for direct expression of ARF5/MONOPTEROS (MP) by AGL15 with Zheng et al. (2009) data showing a significant reduction in mRNA in the *agl15 agl18* double mutant, although the *35S:AGL15* did not show increased accumulation of this transcript. *ARF5*, when present as a loss-of-function, had the largest effect on SE efficiency and productivity, but overexpressing this gene did not produce any SE for the few explants that could be obtained; again, indicating that restrictions on auxin response may be important for SE (Wójcikowska and Gaj, 2017). ARF3/ETTIN is repressed by AGL15, and transcript accumulation of this ARF is negatively correlated with time in SE culture (Wójcikowska and Gaj, 2017). Therefore, some auxin responsive elements behave as would be believed consistent with SE. We comment on how auxin interacts with other select PGRs below.

### AGL15 and GA

Auxin signaling leads to GA accumulation in some tissues by activation of GA biosynthetic genes and/or GA catabolic gene deactivation (Weiss and Ori, 2007). Therefore, it is possible that repression of the auxin response by *35S:AGL15* may reduce the biologically active GA, thereby promoting SE. Our prior work corroborated this hypothesis as we found that AGL15 directly up regulates a GA catabolism gene *GA2ox6* in both Arabidopsis and soybean (Zheng et al., 2009, 2016). A reduction of biologically active GA was also observed in *35S*:*AGL15* compared with the control (Wang et al., 2004). In addition, a GA biosynthetic gene, *GA3ox2* (At1g80340) was repressed by AGL15 (Zheng et al., 2009). The accumulation of biologically active GA was inversely correlated with SE in both Arabidopsis and soybean (Wang et al., 2004; Zheng et al., 2016). The addition of a GA biosynthesis inhibitor, paclobutrazol, to the D40 medium promotes SE significantly in soybean (Zheng et al., 2016), as well as SAM SE in Arabidopsis (Wang et al., 2004). Interestingly, *GA20ox1* (At4g25420), a gene encoding a GA biosynthetic enzyme (Oh et al., 2014), was induced in auxin response and directly correlated with ARF6. Thus, AGL15 also regulates GA accumulation by controlling auxin response. In addition, destabilization of DELLAs occurs in response to GA (Weiss and Ori, 2007). The gibberellin-inactivated REPRESSOR OF GA1-3 (RGA), a DELLA regulatory protein, prevents the binding of ARF6 to DNA. GA perception leads to the degradation of RGA, thereby enabling ARF6 to bind target genes and cause auxin-responsive gene regulation. Another DELLA protein, GIBBERELLIC ACID INSENSITIVE (GAI), is a putative direct expressed target of ARF6 (Oh et al., 2014). Based on microarray results, both the DELLA encoding genes *GmRGA* and *GmGAI* showed increased transcript accumulation in the 35Spro:*GmAGL15* on 2,4-D (Zheng et al., 2013a,b). Therefore, we found a complex and interesting interaction between hormones that would also include feedback mechanisms.

### AGL15 and Ethylene

The effects of the gaseous hormone ethylene on plants have been well-studied on plant growth, development, and stress responses. The interaction between different hormonal and developmental signals is critical in this response. According to our prior work, (Gm)AGL15 impacts ethylene biosynthesis and perception, which is relevant to SE (Zheng et al., 2013a). Auxin-ethylene crosstalk reveals that they interact in a complex coregulatory manner (Stepanova et al., 2008; Vandenbussche et al., 2012). Auxin and ethylene impact each other's accumulation with 2,4-D treatment leading to an increase in ethylene accumulation (Raghavan et al., 2006; Stepanova et al., 2008; Vandenbussche et al., 2012). Conversely, *TRYPTOPHAN AMINOTRANSFERASE RELATED2* (*TAR2)*, that encodes an α class of pyridoxal-50-phosphate (PLP) dependent enzymes (Stepanova et al., 2008), was a potential direct target of AGL15 and is expressed in response to AGL15 (Zheng et al., 2009). *TAR2* is involved in ethylene response and encodes an enzyme involved in auxin biosynthesis (Ma et al., 2014).

In soybean, GmAGL15 was found to increase transcript accumulation from several ethylene biosynthetic genes and *35S:GmAGL15* tissue has significantly higher ethylene accumulation than the control. Genes involved in ethylene signaling were often regulated in a consistent manner in response to AGL15 in soybean and Arabidopsis (Zheng et al., 2013a). For example, genes encoding potential orthologs of MtSERF1 in Arabidopsis (one gene; At5g61590) and soybean (two genes; Glyma20g16920 and Glyma10g24360), were found to be directly upregulated targets of (Gm)AGL15. MtSERF1 is a *Medicago truncatula* ethylene response factor subfamily B-3 of APETALA2/ETHYLENE RESPONSE FACTOR (AP2/ERF) transcription factors that was shown to be required for somatic embryogenesis. This gene is induced by ethylene and responds to auxin and cytokinin (Mantiri et al., 2008). The Arabidopsis ortholog of MtSERF1 was further demonstrated to be relevant for SAM SE (Zheng et al., 2013a). These studies allowed non-transgenic approaches to facilitate SE in both Arabidopsis and soybean by manipulating ethylene biosynthesis and perception. A precursor to ethylene, 1-aminocyclopropane-1-carboxylic-acid (ACC), enhanced SE in both species, whereas inhibitors of ethylene synthesis or perception decreased SE.

Depending on the context, the ethylene-GA crosstalk may be synergistic or antagonistic (Weiss and Ori, 2007). Interestingly, transcript accumulation of both the soybean and Arabidopsis *SERF1* showed inverse correlation with GA, supporting an antagonistic interaction in SE. In addition, antagonistic interaction may also act by via stabilizing the GA repressor DELLA proteins by ethylene signaling via EIN3 and CTR1 (Zheng et al., 2013a). Finally, Saptari and Susila (2019) demonstrated that *AGL15, FUS3*, and *BBM* show increased transcript accumulation in response to ethylene via EIN3, whereas Zheng et al. (2013a), showed a positive correlation between ethylene and GmAGL15, GmAGL18, GmFUS3, and GmABI3 in soybean. Conversely, these genes showed a general negative correlation with biologically active GA (Zheng et al., 2016).

### AGL15 and Brassinosteroids

While Brassinosteroids (BR) have been reported to enhance SE (at least in combination with cytokinin: CK—Chone et al., 2018), a recent publication reports on direct interactions between AGL15, BR signaling, and SE (Ruan et al., 2021). This paper documents experiments showing that BR promotes the embryo to seedling transition, at least in part through repression of *AGL15* expression. Two positive regulators of BR signaling, BRI1-EMS-SUPPRESSOR 1 (BES1), and BRASSINAZOLE-RESISTANT 1 (BZR1), were found to bind directly to *AGL15's* promoter region. Furthermore, inhibition of BR biosynthesis chemically or through mutants can promote SE development on seedlings of the single mutant *val1* that normally only shows this phenotype in combination with *val2* mutants (discussed below), whereas dominant forms of *bes1-D* and *bzr1-1D* can rescue normal seedling development. AGL15 is involved in the production of the SEs as shown by double and triple mutants with an *agl15* loss-of-function mutant. Ruan et al. (2021) hypothesized that BES1 and BZR1 may also directly regulate GA biosynthetic genes, increasing bioactive GA levels and thereby removing negative interaction of BES1/BZR1 by the DELLA proteins.

Interestingly, Ruan et al. (2021) also show that wild type seedlings treated with BR biosynthesis inhibitors have darker green, downward curling leaves that look similar to seedings overexpressing AGL15 (Fernandez et al., 2000). AGL15 binds to the promoter regions of *BZR1* and *BES1* (Supplementary Figure 1) and at least for *BZR1*, represses transcript accumulation. Thus, there may be an intriguing feedback loop between BR signaling, AGL15, and GA to control the transition from seed to seedling.

## Agl15 Directly Binds Genes Encoding Micro-Rnas

MicroRNA (miRNA) function is critical during embryogenesis through various cellular, physiological, and developmental processes. MiRNAs target transcripts from genes that include those with a regulatory role such as TFs that can mediate stress adaptation, developmental regulation, and hormone response (Jones-Rhoades et al., 2006; Jin et al., 2020). MiRNAs are single-stranded RNA molecules of 21– 24 nucleotides that post-transcriptionally regulate gene expression (Bartel, 2009; Rubio-Somoza and Weigel, 2011). The mechanism of miRNA action is either by targeting mRNA for cleavage or by inhibiting translation (Yu et al., 2017). RNA cleavage is mediated by recognizing the nearly perfect complementary sites (Llave et al., 2002; Tang et al., 2003) and involves numerous proteins that are associated with the RNA-induced silencing complex (RISC).

Briefly, *MIR* genes encoding microRNAs are transcribed by RNA pol II forming primary miRNAs (pri-miRNAs) with a characteristic hairpin structure that are processed by a complex that includes DICER-LIKE1 (DCL1) to generate a pre-miRNA. This pre-miRNA duplex structure is further processed, producing the mature single stranded miRNA, which is incorporated by ARGONAUTE1 (AGO1) to form an active RISC (Baumberger and Baulcombe, 2005). This complex is involved in the identification of the target transcripts that are complementary to the miRNA sequence (Rhoades et al., 2002; Kidner and Martienssen, 2005).

There are excellent resources on the prominence of microRNAs, and their functions on plant development (Bartel, 2009; Rubio-Somoza and Weigel, 2011; Wójcikowska et al., 2020; Gyawali et al., 2021; Ma et al., 2021). Several studies highlight the crucial role of miRNA in plant development, especially focusing on embryogenesis, including SE (Armenta-Medina et al., 2017; Liu et al., 2018; Song et al., 2019; Alves et al., 2020; Wójcikowska et al., 2020). Here, we emphasize how AGL15, in addition to targeting protein-encoding genes, also regulates *MIR* genes. Some miRNAs repress key embryo identity genes *LEC2* and FUS3 that encode products involved in embryo maturation (Willmann et al., 2011). Other miRNAs in Arabidopsis play roles in cell-specific gene expression programs in response to spatial and temporal signals, to prevent precocious gene expression, and to enable pattern formation (Nodine and Bartel, 2010; Plotnikova et al., 2019). In the early eight-cell stage of zygotic embryogenesis, DCL1 is essential for cell differentiation. Similarly, *dcl* mutants in somatic explants are unable to initiate embryogenic induction *in vitro* (Wójcik and Gaj, 2016). Another large-scale study showed the dynamics and functions of miRNA during Arabidopsis embryogenesis (Plotnikova et al., 2019). The group applied a high-throughput sequencing technique to profile hundreds of miRNAs and their targets throughout embryogenesis. The findings highlight that miRNAs dynamically cleave and repress at least 59 transcripts, including 30 encoding transcription factors belonging to eight different families (Plotnikova et al., 2019). Other studies demonstrated that mutant lines of genes encoding miR160, miR170/171, or miR319 result in incorrect divisions in the embryo and have abnormal cotyledon development, suggesting that the corresponding miRNA activities are required for embryo morphogenesis (Palatnik et al., 2003; Mallory et al., 2005; Liu et al., 2010; Takanashi et al., 2018).

Szyrajew et al. (2017), examined the expression of 190 *miRNA* genes during Arabidopsis somatic embryogenesis and found that 98% of the *MIR* genes were active during SE, with 64% being differentially expressed during SE. Those differentially expressed during SE included *miR156, miR157, miR159, miR160, miR164, miR166, miR169, miR319, miR390, miR393, miR396*, and *miR398*. These miRNAs are well known to have functions associated with phytohormone and stress-related response (Szyrajew et al., 2017). One example, miR393, regulates the expression of auxin receptors during early SE. The authors suggest that increased miR393, resulting in reduced auxin sensitivity by post-transcriptional regulation of production of auxin receptors, is involved in the transition of somatic cells to embryogenic fate in Arabidopsis (Wójcikowska and Gaj, 2016).

Recently, Nowak et al. (2020) reported how AGL15 negatively regulates the expression of miRNA biogenesis genes through histone acetylation to control the embryogenic reprogramming of somatic cells in Arabidopsis. While AGL15 accumulation showed a positive correlation with transcript accumulation of *pri-miR156h*, the mature miR156h accumulation did not agree with the pri-miRNA and in fact the *agl15agl18* mutant showed increased mature miR156h (and miR156 from other family members) compared to *35S:AGL15*. This indicated a disconnect between production of the pri- and mature miRNA suggesting regulation of miRNA processing components. They found a negative correlation between AGL15 accumulation and transcript accumulation from the miRNA biogenesis genes *DCL1, SERRATE*, and *HUA-ENHANCER1* (*HEN1*). While they did not perform ChIP for AGL15 for these genes, *DCL1* had been identified as a direct target of AGL15 (Zheng et al., 2009) and they did document histone acetylation states in the regulatory regions of these genes, demonstrating increased acetylation in the *agl15agl18* mutant and decreased acetylation in the *35S:AGL15* tissue compared to control for *DCL1* and *SERRATE*. This would be consistent with AGL15 recruitment of histone deacetylase complexes to repress gene expression (discussed further below) (Hill et al., 2008). Neither *SERRATE* nor *HEN1* were found as targets in our ChIP studies (Zheng et al., 2009; Paul et al., 2021).

Our results found that AGL15 directly regulates miR167a, with subsequent impacts on targets *ARF6* and *ARF8*, with consequences for SAM SE. These results are discussed under the section on auxin. Here we present the set of miRNA encoding genes with regulatory regions associated with AGL15 (Table 3). All of the miRNAs mentioned in Szyrajew et al. (2017) have members bound by AGL15. Because the miRNAs were not represented on the microarray, we looked at response of targets of these miRNAs as predicted by TarDB (http://www.biosequencing.cn/TarDB/guide/guide.html) (Liu et al., 2021). Response of the targets and annotation is provided in Supplementary Table 2.

**Table 3:** miRNAs that have associated regions bound by AGL15 (based on ChIP-chip and/or ChIP-seq). Included are predicted targets from TarDB (Liu et al., 2021) and notations of involvement in SE and/or hormone function.

miRNA associated with AGL15	Predicted targets of the miRNA that show significant response to AGL15 accumulation in *agl15 agl18* mutants and/or *35S:AGL15* 10 d SAM SE (from TarDB: http://www.biosequencing.cn/TarDB/)	Include targets involved in hormone response; miRNA involved in SE (Szyrajew et al., 2017; Jin et al., 2020)
miR156 (a,c,d,e)	AT1G11190,AT1G11840,AT1G48760,AT1G63080,AT2G27050,AT3G05430, AT3G10910,AT3G17330,AT4G34990,AT4G35620,AT4G36650,AT1G71400, AT2G34190,AT3G22160,AT5G43270,AT5G43460,AT5G50570,AT5G63850, AT4G30790	Ethylene;Yes
miR157 (a,b,)	AT3G22790,AT5G50570,AT1G30460,AT2G13290,AT4G16860,AT5G50570	Ethylene; Yes
miR158a	AT1G17145,AT1G62930,AT1G65810,AT2G22300,AT2G46590,AT3G01040, AT3G09850,AT3G12010,AT3G23690,AT4G30690, AT4G37740,AT5G35670, AT5G38840,AT5G40340,AT5G43460,AT5G52060,AT5G53220	
miR159 (a,b,)	AT1G08030,AT1G21060,AT1G51780,AT3G44690,AT3G53810,AT4G12240, AT1G04700,AT1G08030,AT1G08550,AT1G30210,AT1G35460,AT1G63130, AT1G78610,AT3G10300,AT3G12550,AT3G28150,AT3G53160,AT4G23950, AT5G04460, AT5G06100,AT5G09670,AT5G12100	ABA; Yes
miR160a	AT1G52620,AT1G79180,AT4G18930,AT4G20430,AT5G43270	Auxin; Yes
miR164b	AT1G76580,AT2G26690,AT4G28550,AT5G07680,AT5G62230	Yes
miR165a	AT1G47530,AT1G58200,AT4G39150,AT5G50570,AT5G67570	
miR166 (a,b,c,d,e,g)	AT1G30490,AT1G61210,AT5G01030,AT1G30490,AT3G16785,AT5G01030, AT1G30490, AT1G30490,AT1G30490,AT5G35670,AT5G65970,AT1G30490	Auxin; Yes
miR167a	AT2G34530,AT3G59470,AT4G30130,AT5G02940,AT5G19950,AT5G49990	Auxin
miR168 (a,b)	AT1G17760,AT2G02470,AT3G22160,AT1G48410,AT2G23130	
miR169 (a,b,c,f)	AT1G72830,AT2G01530,AT2G30600,AT3G27700,AT4G27710,AT4G36710, AT5G12470,AT1G72830,AT5G38840, AT1G72830,AT5G38840,AT1G72830, AT3G15950,AT3G61250,AT4G18890,AT5G62680	Yes
miR170	AT4G17370,AT5G16390,AT5G65970	
miR172a	AT1G12270,AT1G28190,AT1G71400,AT2G34190,AT2G47460,AT3G07060, AT3G23330,AT4G16860,AT4G36650, AT5G02710,AT5G16260,AT5G17760, AT5G63850	
miR173	AT3G58780	
miR319 (a,c)	AT1G30210,AT1G04700,AT1G53230,AT3G06500	Yes
miR390 (a,b)	AT3G04110,AT3G13430,AT5G05180,AT5G62230,AT1G55610,AT2G02470, AT2G33730,AT5G62230	Auxin; Yes
miR393a	AT1G15290,AT1G48410,AT2G46340,AT3G54280,AT4G26120	Auxin; Yes
miR394 (a,b)	AT1G06580,AT1G19630,AT1G62910,AT3G07770,AT3G15840,AT3G48460, AT3G48460,AT3G49240,AT4G20430,AT5G51100,AT5G56950,AT3G48460, AT4G20430,AT4G20430,AT5G07870,AT5G09670,AT5G51100,AT5G56950	
miR396b	AT1G12820AT1G28010AT1G30210AT1G30490AT1G52500AT1G53AT1G55610, AT1G60140,AT1G61370,AT1G62590,AT1G74790,AT1G76810,AT2G23130, AT2G33730,AT2G40840,AT3G01370,AT3G03790,AT3G16730,AT3G16785, AT3G17090,AT3G24310,AT3G54350,AT3G59420,AT4G19050,AT4G19440, AT4G20440,AT4G30080,AT4G30130,AT4G32710,AT5G01030,AT5G12900, AT5G39610,AT5G42830,AT5G46110,AT5G61440	Yes
miR398c	AT1G30460, AT5G19950	
miR399 (a,c)	AT3G60040,AT3G62980,AT5G42880,AT1G71350,AT2G19470,AT2G33730, AT2G33770,AT2G33770,AT3G06500,AT3G15130,AT3G59420,AT5G51220, AT5G60890	
miR400	AT1G07590,AT1G34670,AT1G48780,AT1G76870,AT2G13290,AT2G19470, AT2G28550,AT2G43970,AT4G26680,AT5G14550,AT5G39710,AT5G41400, AT5G53950,AT5G63490	
mir403	AT4G31460,AT5G39900	
miR858a	AT1G50700,AT1G53230,AT1G65540,AT1G79640,AT3G10120,AT3G59470, AT3G61570,AT5G46560,AT5G59900,AT5G66420	

## Agl15 and Potential Links To Epigenetic Regulation of Somatic Embryogenesis

Epigenetic modifications are crucial in development processes. The structure of chromatin can be accessible or repressive due to various epigenetic mechanisms like DNA methylation, histone modifications, and chromatin remodeling. These epigenetic mechanisms are dynamic; involved in different plant developmental phases, and in plant adaptation in various environmental stresses. Epigenetic mechanisms could reprogram cell fate and could stabilize cell identity and maintain tissues (Inácio et al., 2018; Hajheidari et al., 2019). *In vitro* culture conditions produce epigenetic variation and are critical factors during SE (De-La-Pena et al., 2015; Kumar and Van Staden, 2017). Many of the loss-of-function mutants in Arabidopsis that produce SE on “seedlings” are mutants defective in epigenetic processes to support the transition from embryo to seedling (reviewed in Tian et al., 2020a). Key embryo-related TFs are inappropriately expressed in these mutants.

### DNA Methylation

One well-studied epigenetic modification is DNA methylation and this has been found to control gene expression essential during early somatic embryogenesis (Stricker et al., 2017; Wójcikowska et al., 2020). DNA methylation involves the addition of a methyl group to the 5′ position of the pyrimidine ring of cytosine. In plants, methylation can occur in CpG islands (Gruenbaum et al., 1981); CpHpG and CpHpHp (where H is any nucleotide except G; (Feng et al., 2010). DNA methylation differs at each stage of SE (De-La-Pena et al., 2015). Several reports have indicated that embryogenic cultures/explants and DNA methylation have an inverse relationship. For example, early somatic cells of leaf explants in *Coffea canephora* show a level of methylated cytosines of about 23.7%, but later stages have higher levels of DNA methylation (Nic-Can et al., 2013). The dedifferentiating Arabidopsis leaf protoplast has been reported to have changes in DNA methylation (Avivi et al., 2004). Auxin's role in inducing epigenetic regulation of SE has been recently reviewed (Wójcik et al., 2020). A recent study shows hypermethylation in the promoters of TFs that are associated with SE in response to auxin treatment (Grzybkowska et al., 2020). They confirmed exogenous auxin substantially affected both the expression and methylation patterns, resulting in control of embryo-related targets *LEC1, LEC2, BBM, WUS*, and *AGL15*, although *AGL15* did not have auxin response elements in the examined region, but did have ethylene response elements (Grzybkowska et al., 2020). They propose that the methylation sensitive ARF, ARF5/MP may be involved in this process.

### Histone Modifications

Apart from DNA methylation, histone modifications have a dynamic function that causes variation of gene expression that is involved in SE. For example, the histone trimethylation mark H3K27me3, generally a repressive mark, is removed from *LEC1* loci, allowing the expression of this TF, and the expression of *BBM1* was related to the increase of both histone marks H3K4me3 and H3K36me2 (generally marks associated with increased transcription) using chromatin immunoprecipitation (ChIP) assays (Nic-Can et al., 2013; Borg et al., 2020). Another technique called immunolocalization was used in *Brassica napus* to evaluate the levels of H3K9me2 that was low in microspores before the induction of SE; however it showed increases during later stages of SE. In contrast to methylation, it was observed that the levels of acetylation of H3 and H4 (H3Ac and H4Ac) were more abundant in microspores before SE induction, suggesting it might increase transcriptional activity and regulate cellular reprogramming and embryo development (Rodríguez-Sanz et al., 2014). Similarly, in *C. canephora*, a decrease in the DNA methylation level has been related to a decrease of H3K9me2 and H3K27me3 during SE (Nic-Can et al., 2013).

One of the interesting groups of proteins related to histone methylation marks that are involved in regulating plant cellular reprogramming are the Polycomb-group (PcG). (reviewed in Duarte-Aké et al., 2019). The PcG proteins are classified into two complexes, POLYCOMB REPRESSIVE COMPLEX 1 and 2 (PRC1 and PRC2), which are involved in repression of genes via histone methylation (Grossniklaus and Paro, 2014; Mozgová et al., 2015). PRC2- mediated histone methylation represses embryo maturation programs during vegetative development in Arabidopsis, where cell fate could reset and SE could occur when PRC2 depleted tissues are treated with hormone (Mozgová et al., 2017). *LEC1, LEC2, FUS3, AGL15, PLT*, and *WOXs*, which have regulatory roles in somatic cell differentiation and SE induction are shown to be targeted by PRC2 in different plants (Ikeuchi et al., 2015; Orłowska et al., 2017; Rose, 2019).

A recent study found that HSI2/VAL1 silences AGL15 to regulate the development transition from seed maturation to vegetative phase in Arabidopsis (Chen et al., 2018). *VIVIPAROUS1/ABI3-LIKE* (VAL1) and VAL2, are also known as HIGH-LEVEL EXPRESSION OF SUGAR INDUCIBLE GENE2 (HSI2) and HSI2-LIKE1 (HSL1), respectively. VAL genes encode B3 domain transcription factors similar to LEC2, FUS3, and ABI3 (Suzuki et al., 2007), but also contain other conserved domains including a plant homeodomain (PHD), a cysteine-and-tryptophan residue containing (CW) domain, and an EAR motif that is involved in recruiting histone deacetylation complexes. These domains are involved in epigenetic regulation, and HSI2/VAL1 recruits PRC2 components. During the transition to seedling development, HSI2/VAL1 is required for the transcriptional silencing of *LAFL* and *AGL15* genes, but further work demonstrated that while this protein interacts directly with regulatory regions of AGL15, no such interaction was found for the *LAFL* genes (Chen et al., 2018). While a binding site for B3 domains are involved in silencing *AGL15*, a mutation specifically within the PHD domain interferes with H3K27me3 at *AGL15* and also prevents silencing, and this involved recruitment of PRC2 components (Veerappan et al., 2014; Chen et al., 2018).

Another mode of histone modification is acetylation where lysine residues on the N-terminal tails of histones undergo acetylation, which causes increased DNA accessibility for TF binding. Histone acetyltransferases (HATs), and histone deacetylases (HDACs) are the complexes that control the acetylation state (Steunou et al., 2014; Lee and Grant, 2019). HDAC6 and HDAC19 are two important deacetylases that are involved in SE where a double knockdown generates SE on seedlings. AGL15 has been found to recruit HDA19 via interact with TOPLESS (TPL) and TOPLESS-RELATED PROTEIN2 (TPR2) (Causier et al., 2012), and SIN3 ASSOCIATED POLYPEPTIDE P18 (SAP18; Hill et al., 2008).

## Discussion and Future Directions

While AGL15 has a role in promoting SE, it is not to the extent as found for some TFs, such as LEC1, LEC2, and BBM, possibly indicating needed protein complex formation with other proteins. However, the interactions between AGL15 and these other factors is fascinating, as is the fact that other factors directly control AGL15 to facilitate the transition from embryo to seedling. Many questions obviously remain. Why does AGL15 appear to limit auxin responses and possibly be involved in negative regulation of AGP signaling? These observations may point to different roles of cells involved in producing SEs. Whereas, some cells produce the somatic embryos, others in the culture are equally important to provide what has been referred to as “nursing” functions. Current technologies allow marking and sorting of cells. An intriguing avenue of investigation would be characterizing AGL15 function in the cells that make SE compared to the support tissues. The observations on GmEP3 at different timepoints of soybean SE culture emphasize the need for examination of more timepoints during the SE process. It is quite possible that genes responding to AGL15 in 10 d SAM SE would behave very differently at earlier timepoints. Other hormones are important for SE that have not been examined for relation to AGL15 function. These include BR, CK and ABA. Notably, genes in these GO categories are overrepresented among AGL15 targets (Figure 5). Finally, comparison of gene regulation in developmental processes in different contexts will be important to tease out commonalities and determine other context dependent factors. As an example, Nowak et al. (2020) documented an inverse correlation between AGL15 and transcripts from miRNA biogenesis genes, indicating repression, direct or indirect of these genes by AGL15. However, data from Zheng et al. (2009), showed decreased transcript accumulation for *DCL1, HEN1*, and *SERRATE* in the *agl15agl18* mutant (non-significant in the *35S:AGL15* compared to control), suggesting expression of these genes. Zheng et al. (2009) used an SAM SE system, whereas Nowak et al. (2020) used immature zygotic embryos as explants, perhaps explaining these and other differences.

Molecular breeding has become a significant tool to improve crop production in a shorter time period, and efficient somatic embryogenesis is a valuable technique in regenerating such crops. Advances in gene editing techniques like CRISPR/Cas9 created the potential to improve crop traits without regulatory concerns that apply to traditional transgenics (once the Cas9 and guide RNAs are segregated, at least in some countries; Grossman, 2019; Eriksson et al., 2019). However, cells engineered for particular traits must be able to exhibit pluripotency and produce a plant to be useful. As yet, it is not well understood why some plants regenerate well, poorly or not at all via either SE or organogenesis. Even particular cultivars of a species can be recalcitrant to these processes. We and others have found that AGL15 is a tool to enhance regeneration via SE, at least in dicots. For example, plant transformation in soybean is accelerated when overexpression of AGL15 enhances somatic embryogenesis, which could be utilized for producing elite cultivars. Similarly, AGL15 could provide a vital role in scaling up production of valuable crops such as coffee through propagation and genetic transformation (Etienne et al., 2018; Xu et al., 2018). Other crops could benefit from examining *AGL15* expression in their species, and possibly enhancing transformation strategies via better regeneration by SE.

## Data Availability Statement

The original contributions presented in the study are included in the article/Supplementary Material, further inquiries can be directed to the corresponding author/s.

## Author Contributions

All authors contributed to writing the manuscript. All authors contributed to the article and approved the submitted version.

## Funding

This work was supported by the National Science Foundation (grant no. IOS-1656380 to SP) and by the National Institute of Food and Agriculture, U.S. Department of Agriculture, Hatch project (SP) under accession number 1013409.

## Conflict of Interest

The authors declare that the research was conducted in the absence of any commercial or financial relationships that could be construed as a potential conflict of interest.

## Publisher's Note

All claims expressed in this article are solely those of the authors and do not necessarily represent those of their affiliated organizations, or those of the publisher, the editors and the reviewers. Any product that may be evaluated in this article, or claim that may be made by its manufacturer, is not guaranteed or endorsed by the publisher.

## References

[B1] Alves A. Rodrigues A. S. Miguel C. (2020). “microRNAs in plant embryogenesis,” in Plant microRNAs. Concepts and Strategies in Plant Sciences, eds C. Miguel, T. Dalmay, and I. Chaves (Cham: Springer), 99–120. 10.1007/978-3-030-35772-6_6

[B2] Armenta-Medina A. Lepe-Soltero D. Xiang D. Datla R. Abreu-Goodger C. Gillmor C. S. (2017). *Arabidopsis thaliana* miRNAs promote embryo pattern formation beginning in the zygote. Dev. Biol. 431, 145–151. 10.1016/j.ydbio.2017.09.009 28912016

[B3] Avivi Y. Morad V. Ben-Meir H. Zhao J. Kashkush K. Tzfira T. . (2004). Reorganization of specific chromosomal domains and activation of silent genes in plant cells acquiring pluripotentiality. Dev. Dyn. 230, 12–22. 10.1002/dvdy.20006 15108305

[B4] Awasthi P. Sharma V. Kaur N. Kaur N. Pandey P. Tiwari S. (2017). Genome-wide analysis of transcription factors during somatic embryogenesis in banana (Musa spp.) cv. Grand Naine. PLoS ONE 12, e0182242. 10.1371/journal.pone.0182242 28797040 PMC5552287

[B5] Bartel D. P (2009). MicroRNAs: target recognition and regulatory functions. Cell 136, 215–233. 10.1016/j.cell.2009.01.002 19167326 PMC3794896

[B6] Baumberger N. Baulcombe D. (2005). Arabidopsis ARGONAUTE1 is an RNA slicer that selectively recruits microRNAs and short interfering RNAs. Proc. Natl. Acad. Sci. U. S. A. 102, 11928–11933. 10.1073/pnas.0505461102 16081530 PMC1182554

[B7] Bidabadi S. S. Jain S. M. (2020). Cellular, molecular, and physiological aspects of *in vitro* plant regeneration. Plants-Basel 9, 702. 10.3390/plants9060702 32492786 PMC7356144

[B8] Borg M. Jacob Y. Susaki D. Leblanc C. Buendía D. Axelsson E. . (2020). Targeted reprogramming of H3K27me3 resets epigenetic memory in plant paternal chromatin. Nature Cell Biol. 22, 621–629. 10.1038/s41556-020-0515-y 32393884 PMC7116658

[B9] Boutilier K. Offringa R. Sharma V. K. Kieft H. Ouellet T. Zhang L. . (2002). Ectopic expression of BABY BOOM triggers a conversion from vegetative to embryonic growth. Plant Cell 14, 1737–1749. 10.1105/tpc.001941 12172019 PMC151462

[B10] Braybrook S. A. Harada J. J. (2008). LECs go crazy in embryo development. Trends Plant Sci. 13, 624–630. 10.1016/j.tplants.2008.09.008 19010711

[B11] Busch W. Miotk A. Ariel F. D. Zhao Z. Forner J. Daum G. . (2010). Transcriptional control of a plant stem cell niche. Dev. Cell 18, 849–861. 10.1016/j.devcel.2010.03.012 20493817

[B12] Causier B. Ashworth M. Guo W. Davies B. (2012). The TOPLESS interactome: a framework for gene repression in Arabidopsis. Plant Physiol. 158, 423–438. 10.1104/pp.111.186999 22065421 PMC3252085

[B13] Chen F. Zhang X. T. Liu X. Zhang L. S. (2017). Evolutionary analysis of MIKCc-type MADS-box genes in gymnosperms and angiosperms. Front. Plant Sci. 8, 895. 10.3389/fpls.2017.00895 PMC5447709 28611810

[B14] Chen N. Veerappan V. Abdelmageed H. Kang M. Allen R. D. (2018). HSI2/VAL1 silences AGL15 to regulate the developmental transition from seed maturation to vegetative growth in Arabidopsis. Plant Cell 30, 600–619. 10.1105/tpc.17.00655 29475938 PMC5894832

[B15] Chone R. M. S. Rocha D. I. Monte-Bello C. C. Pinheiro H. P. Dornelas M. C. Haddad C. R. B. . (2018). Brassinosteroid increases the cytokinin efficiency to induce direct somatic embryogenesis in leaf explants of *Coffea arabica* L. (Rubiaceae). Plant Cell Tissue Organ Cult. 135, 63–71. 10.1007/s11240-018-1443-4

[B16] Clough S. J. Bent A. F. (1998). Floral dip: a simplified method for Agrobacterium-mediated transformation of *Arabidopsis thaliana*. Plant J. 16, 735–743. 10.1046/j.1365-313x.1998.00343.x 10069079

[B17] De-La-Pena C. Nic-Can G. I. Galaz-Avalos R. M. Avilez-Montalvo R. Loyola-Vargas V. M. (2015). The role of chromatin modifications in somatic embryogenesis in plants. Front. Plant Sci. 6, 635. 10.3389/fpls.2015.00635 PMC4539545 26347757

[B18] Domon J. M. Neutelings G. Roger D. David A. David H. (2000). A basic chitinase-like protein secreted by embryogenic tissues of *Pinus caribaea* acts on arabinogalactan proteins extracted from the same cell lines. J. Plant Physiol. 156, 33–39. 10.1016/S0176-1617(00)80269-2

[B19] Duarte-Aké F. Nic-Can G. De-La-Peña C. (2019). “Somatic embryogenesis: polycomb complexes control cell-to-embryo transition,” in Epigenetics in Plants of Agronomic Importance: Fundamentals and Applications, eds R. Alvarez-Venegas, C. De-la-Pena, and J. Casas-Mollano (Cham: Springer), 339–354. 10.1007/978-3-030-14760-0_13

[B20] Emons M. C (1994). Somatic embryogenesis: cell biological aspects. Acta Botanica Neerlandica 43, 1–14. 10.1111/j.1438-8677.1994.tb00729.x

[B21] Eriksson D. Kershen D. Nepomuceno A. Pogson B. J. Prieto H. Purnhagen K. . (2019). A comparison of the EU regulatory approach to directed mutagenesis with that of other jurisdictions, consequences for international trade and potential steps forward. New Phytol. 222, 1673–1684. 10.1111/nph.15627 30548610

[B22] Etienne H. Breton D. Breitler J. C. Bertrand B. Dechamp E. Awada R. . (2018). Coffee somatic embryogenesis: how did research, experience gained and innovations promote the commercial propagation of elite clones from the two cultivated species? FIPS 9, 1630. 10.3389/fpls.2018.01630 PMC6240679 30483287

[B23] Fehér A (2015). Somatic embryogenesis—stress-induced remodeling of plant cell fate. BBA-Gene Reg. Mech. 1849, 385–402. 10.1016/j.bbagrm.2014.07.005 25038583

[B24] Fehér A (2019). Callus, dedifferentiation, totipotency, somatic embryogenesis: what these terms mean in the era of molecular plant biology? Front. Plant Sci. 10, 536. 10.3389/fpls.2019.00536 PMC6524723 31134106

[B25] Feng S. Jacobsen S. E. Reik W. (2010). Epigenetic reprogramming in plant and animal development. Science 330, 622–627. 10.1126/science.1190614 21030646 PMC2989926

[B26] Fernandez D. E. Heck G. R. Perry S. E. Patterson S. E. Bleecker A. B. Fang S.-C. (2000). The embryo MADS domain factor AGL15 acts postembryonically: inhibition of perianth senescence and abscission via constitutive expression. Plant Cell 12, 183–197. 10.1105/tpc.12.2.183 10662856 PMC139757

[B27] Gaj M. D. Trojanowska A. Ujczak A. Medrek M. Koziol A. Garbaciak B. (2006). Hormone-response mutants of *Arabidopsis thaliana* (L.) Heynh. impaired in somatic embryogenesis. Plant Growth Reg. 49, 183–197. 10.1007/s10725-006-9104-8

[B28] Ghadirzadeh-Khorzoghi E. Jahanbakhshian-Davaran Z. Seyedi S. M. (2019). Direct somatic embryogenesis of drought resistance pistachio (*Pistacia vera* L.) and expression analysis of somatic embryogenesis-related genes. South Afr. J. Bot. 121, 558–567. 10.1016/j.sajb.2019.01.023

[B29] Goodstein D. M. Shu S. Howson R. Neupane R. Hayes R. D. Fazo J. . (2012). Phytozome: a comparative platform for green plant genomics. Nucl. Acids Res. 40, D1178–D1186. 10.1093/nar/gkr944 22110026 PMC3245001

[B30] Grafi G (2009). The complexity of cellular dedifferentiation: implications for regenerative medicine. Trends Biotech. 27, 329–332. 10.1016/j.tibtech.2009.02.007 19395104

[B31] Grafi G. Chalifa-Caspi V. Nagar T. Plaschkes I. Barak S. Ransbotyn V. (2011). Plant response to stress meets dedifferentiation. Planta 233, 433–438. 10.1007/s00425-011-1366-3 21312042

[B32] Gramzow L. Theissen G. (2010). A hitchhiker's guide to the MADS world of plants. Genome Biol. 11, 214–214. 10.1186/gb-2010-11-6-214 20587009 PMC2911102

[B33] Grossman M. R (2019). “New plant breeding technologies: US Department of Agriculture policy,” in Functional Field of Food Law: Reconciling the Market and Human Rights, eds A. Urazbaeva, A. Szajkowska, B. Wernaart, N. T. Franssens, and R. S. Vaskoska (Wageningen: Wageningen Academic Publications), 397–416. 10.3920/978-90-8686-885-8_26

[B34] Grossniklaus U. Paro R. (2014). Transcriptional silencing by polycomb-group proteins. Cold Spring Harb. Perspect. Biol. 6, a019331. 10.1101/cshperspect.a019331 25367972 PMC4413232

[B35] Grover A (2012). Plant chitinases: genetic diversity and physiological roles. Critic. Rev. Plant Sci. 31, 57–73. 10.1080/07352689.2011.616043

[B36] Gruenbaum Y. Naveh-Many T. Cedar H. Razin A. (1981). Sequence specificity of methylation in higher plant DNA. Nature 292, 860–862. 10.1038/292860a0 6267477

[B37] Grzybkowska D. Nowak K. Gaj M. D. (2020). Hypermethylation of auxin-responsive motifs in the promoters of the transcription factor genes accompanies the somatic embryogenesis induction in Arabidopsis. Int. J. Mol. Sci. 21, 6849. 10.3390/ijms21186849 32961931 PMC7555384

[B38] Guan Y. Li S.-G. Fan X.-F. Su Z.-H. (2016). Application of somatic embryogenesis in woody plants. Front. Plant Sci. 7, 938. 10.3389/fpls.2016.00938 PMC4919339 27446166

[B39] Guilfoyle T. J. Hagen G. (2012). Getting a grasp on domain III/IV responsible for auxin response factor–IAA protein interactions. Plant Sci. 190, 82–88. 10.1016/j.plantsci.2012.04.003 22608522

[B40] Gyawali B. Barozai M. Y. K. Aziz A. N. (2021). Comparative expression analysis of microRNAs and their targets in emerging bio-fuel crop sweet sorghum (*Sorghum bicolor* L.). Plant Gene 26, 100274. 10.1016/j.plgene.2021.100274

[B41] Hajheidari M. Koncz C. Bucher M. (2019). Chromatin evolution-key innovations underpinning morphological complexity. Front. Plant Sci. 10, 454. 10.3389/fpls.2019.00454 PMC6474313 31031789

[B42] Harding E. W. Tang W. Nichols K. W. Fernandez D. E. Perry S. E. (2003). Expression and maintenance of embryogenic potential is enhanced through constitutive expression of *AGAMOUS-Like 15*. Plant Physiol. 133, 653–663. 10.1104/pp.103.023499 14512519 PMC219041

[B43] Hayashi K.-I (2012). The interaction and integration of auxin signaling components. Plant Cell Physiol. 53, 965–975. 10.1093/pcp/pcs035 22433459

[B44] Heck G. R. Perry S. E. Nichols K. W. Fernandez D. E. (1995). AGL15, a MADS domain protein expressed in developing embryos. Plant Cell 7, 1271–1282. 10.1105/tpc.7.8.1271 7549483 PMC160950

[B45] Hesami M. Naderi R. Tohidfar M. (2020). Introducing a hybrid artificial intelligence method for high-throughput modeling and optimizing plant tissue culture processes: the establishment of a new embryogenesis medium for chrysanthemum, as a case study. Appl. Microbiol. Biotech. 104, 10249–10263. 10.1007/s00253-020-10978-1 33119796

[B46] Hill K. Wang H. Perry S. E. (2008). A transcriptional repression motif in the MADS factor AGL15 is involved in recruitment of histone deacetylase complex components. Plant J. 53, 172–185. 10.1111/j.1365-313X.2007.03336.x 17999645

[B47] Horstman A. Fukuoka H. Muino J. M. Nitsch L. Guo C. H. Passarinho P. . (2015). AIL and HDG proteins act antagonistically to control cell proliferation. Development 142, 454–464. 10.1242/dev.117168 25564655

[B48] Ikeda M. Kamada H. (2005). “Comparison of molecular mechanisms of somatic and zygotic embryogenesis,” in Somatic Embryogenesis, eds A. Mujib and J. Samaj (Berlin: Springer-Verlag), 51–68. 10.1007/7089_027

[B49] Ikeuchi M. Iwase A. Rymen B. Harashima H. Shibata M. Ohnuma M. . (2015). PRC2 represses dedifferentiation of mature somatic cells in Arabidopsis. Nat. Plants 1, 1–7. 10.1038/nplants.2015.89 27250255

[B50] Ikeuchi M. Ogawa Y. Iwase A. Sugimoto K. (2016). Plant regeneration: cellular origins and molecular mechanisms. Development 143, 1442–1451. 10.1242/dev.134668 27143753

[B51] Inácio V. Martins M. T. Graça J. Morais-Cecílio L. (2018). Cork oak young and traumatic periderms show PCD typical chromatin patterns but different chromatin-modifying genes expression. Front. Plant Sci. 9, 1194. 10.3389/fpls.2018.01194 PMC6120546 30210513

[B52] Jha P. Kumar V. (2018). BABY BOOM (BBM): a candidate transcription factor gene in plant biotechnology. Biotech. Lett. 40, 1467–1475. 10.1007/s10529-018-2613-5 30298388

[B53] Jha P. Ochatt S. J. Kumar V. (2020). WUSCHEL: a master regulator in plant growth signaling. Plant Cell Rep. 39, 431–444. 10.1007/s00299-020-02511-5 31984435

[B54] Ji H. K. Jiang H. Ma W. X. Johnson D. S. Myers R. M. Wong W. H. (2008). An integrated software system for analyzing ChIP-chip and ChIP-seq data. Nat. Biotech. 26, 1293–1300. 10.1038/nbt.1505 18978777 PMC2596672

[B55] Jin L. Yarra R. Zhou L. Zhao Z. Cao H. (2020). miRNAs as key regulators via targeting the phytohormone signaling pathways during somatic embryogenesis of plants. 3 Biotech 10, 495. 10.1007/s13205-020-02487-9 33150121 PMC7596142

[B56] Jones-Rhoades M. W. Bartel D. P. Bartel B. (2006). MicroRNAs and their regulatory roles in plants. Annu. Rev. Plant Biol. 57, 19–53. 10.1146/annurev.arplant.57.032905.105218 16669754

[B57] Karlova R. Boeren S. Russinova E. Aker J. Vervoort J. de Vries S. (2006). The Arabidopsis SOMATIC EMBRYOGENESIS RECEPTOR-LIKE KINASE1 protein complex includes BRASSINOSTEROID-INSENSITIVE1. Plant Cell 18, 626–638. 10.1105/tpc.105.039412 16473966 PMC1383638

[B58] Kaul S. Koo H. L. Jenkins J. Rizzo M. Rooney T. Tallon L. J. . (2000). Analysis of the genome sequence of the flowering plant Arabidopsis thaliana. Nature 408, 796–815. 10.1038/35048692 11130711

[B59] Kidner C. A. Martienssen R. A. (2005). The developmental role of microRNA in plants. Curr. Opin. Plant Biol. 8, 38–44. 10.1016/j.pbi.2004.11.008 15653398

[B60] Kumar V. Van Staden J. (2017). New insights into plant somatic embryogenesis: an epigenetic view. Acta Physiol. Plant. 39, 194. 10.1007/s11738-017-2487-5

[B61] Kurczynska E. U. Gaj M. D. Ujczak A. Mazur E. (2007). Histological analysis of direct somatic embryogenesis in *Arabidopsis thaliana* (L.) Heynh. Planta 226, 619–628. 10.1007/s00425-007-0510-6 17406890

[B62] Lee C. Y. Grant P. A. (2019). “Role of histone acetylation and acetyltransferases in gene regulation,” in Toxicoepigenetics, eds S. D. McCullough and D. C. Dolinoy (London: Elsevier), 3–30. 10.1016/B978-0-12-812433-8.00001-0

[B63] Lemoine F. Correia D. Lefort V. Doppelt-Azeroual O. Mareuil F. Cohen- Boulakia S. . (2019). NGPhylogeny.fr: new generation phylogenetic services for non-specialists. Nucleic Acids Res. 47, W260–W265. 10.1093/nar/gkz303 31028399 PMC6602494

[B64] Letunic I. Bork P. (2019). Interactive Tree Of Life (iTOL) v4: recent updates and new developments. Nucleic Acids Res. 47, W256–W259. 10.1093/nar/gkz239 30931475 PMC6602468

[B65] Liu H. Yu H. Tang G. Huang T. (2018). Small but powerful: function of microRNAs in plant development. Plant Cell Rep. 37, 515–528. 10.1007/s00299-017-2246-5 29318384

[B66] Liu J. Liu X. Zhang S. Liang S. Luan W. Ma X. (2021). TarDB: an online database for plant miRNA targets and miRNA-triggered phased siRNAs. BMC Genomics 22, 348. 10.1186/s12864-021-07680-5 33985427 PMC8120726

[B67] Liu X. Huang J. Wang Y. Khanna K. Xie Z. Owen H. A. . (2010). The role of floral organs in carpels, an Arabidopsis loss-of-function mutation in MicroRNA160a, in organogenesis and the mechanism regulating its expression. Plant J. 62, 416–428. 10.1111/j.1365-313X.2010.04164.x 20136729

[B68] Llave C. Xie Z. Kasschau K. D. Carrington J. C. (2002). Cleavage of SCARECROW-like mRNA targets directed by a class of Arabidopsis miRNA. Science 297, 2053–2056. 10.1126/science.1076311 12242443

[B69] Ma W. Y. Li J. J. Qu B. Y. He X. Zhao X. Q. Li B. . (2014). Auxin biosynthetic gene TAR2 is involved in low nitrogen-mediated reprogramming of root architecture in Arabidopsis. Plant J. 78, 70–79. 10.1111/tpj.12448 24460551

[B70] Ma X. Denyer T. Javelle M. Feller A. Timmermans M. C. (2021). Genome-wide analysis of plant miRNA action clarifies levels of regulatory dynamics across developmental contexts. Genome Res. 31, 811–822. 10.1101/gr.270918.120 33863807 PMC8092011

[B71] Magnani E. Jiménez-Gómez J. M. Soubigou-Taconnat L. Lepiniec L. Fiume E. (2017). Profiling the onset of somatic embryogenesis in Arabidopsis. BMC Genomics 18, 998. 10.1186/s12864-017-4391-1 29284399 PMC5747089

[B72] Mallory A. C. Bartel D. P. Bartel B. (2005). MicroRNA-directed regulation of Arabidopsis AUXIN RESPONSE FACTOR17 is essential for proper development and modulates expression of early auxin response genes. Plant Cell 17, 1360–1375. 10.1105/tpc.105.031716 15829600 PMC1091760

[B73] Mantiri F. R. Kurdyukov S. Lohar D. P. Sharopova N. Saeed N. A. Wang X. D. . (2008). The transcription factor MtSERF1 of the ERF subfamily identified by transcriptional profiling is required for somatic embryogenesis induced by auxin plus cytokinin in *Medicago truncatula*. Plant Physiol. 146, 1622–1636. 10.1104/pp.107.110379 18235037 PMC2287338

[B74] Mareri L. Romi M. Cai G. (2019). Arabinogalactan proteins: actors or spectators during abiotic and biotic stress in plants? Plant Biosyst. 153, 173–185. 10.1080/11263504.2018.147352523623239

[B75] Márquez-López R. E. Pérez-Hernández C. Ku-González Á. Galaz-Ávalos R. M. Loyola-Vargas V. M. (2018). Localization and transport of indole-3-acetic acid during somatic embryogenesis in Coffea canephora. Protoplasma 255, 695–708. 10.1007/s00709-017-1181-1 29119309

[B76] Mendez-Hernandez H. A. Ledezma-Rodriguez M. Avilez-Montalvo R. N. Juarez-Gomez Y. L. Skeete A. Avilez-Montalvo J. . (2019). Signaling overview of plant somatic embryogenesis. Front. Plant Sci. 10, 77. 10.3389/fpls.2019.00077 30792725 PMC6375091

[B77] Mozgová I. Köhler C. Hennig L. (2015). Keeping the gate closed: functions of the polycomb repressive complex PRC 2 in development. Plant J. 83, 121–132. 10.1111/tpj.12828 25762111

[B78] Mozgová I. Muñoz-Viana R. Hennig L. (2017). PRC2 represses hormone-induced somatic embryogenesis in vegetative tissue of *Arabidopsis thaliana*. PLoS Genet. 13, e1006562. 10.1371/journal.pgen.1006562 28095419 PMC5283764

[B79] Nic-Can G. I. López-Torres A. Barredo-Pool F. Wrobel K. Loyola-Vargas V. M. Rojas-Herrera R. . (2013). New insights into somatic embryogenesis: LEAFY COTYLEDON1, BABY BOOM1 and WUSCHEL-RELATED HOMEOBOX4 are epigenetically regulated in *Coffea canephora*. PLoS ONE 8, e72160. 10.1371/journal.pone.0072160 23977240 PMC3748027

[B80] Nodine M. D. Bartel D. P. (2010). MicroRNAs prevent precocious gene expression and enable pattern formation during plant embryogenesis. Genes Dev. 24, 2678–2692. 10.1101/gad.1986710 21123653 PMC2994041

[B81] Nowak K. Moronczyk J. Wójcik A. Gaj M. D. (2020). AGL15 controls the embryogenic reprogramming of somatic cells in Arabidopsis through the histone acetylation-mediated repression of the miRNA biogenesis genes. Int. J. Mol. Sci. 21, 6733. 10.3390/ijms21186733 32937992 PMC7554740

[B82] Ochoa-Alejo N. Loyola-Vargas V. M. (2016). Somatic Embryogenesis: Fundamental Aspects and Applications. Springer. 10.1007/978-3-319-33705-0

[B83] Oh E. Zhu J.-Y. Bai M.-Y. Arenhart R. A. Sun Y. Wang Z.-Y. (2014). Cell elongation is regulated through a central circuit of interacting transcription factors in the Arabidopsis hypocotyl. Elife 3, e03031. 10.7554/eLife.03031.025 24867218 PMC4075450

[B84] Orłowska A. Igielski R. Łagowska K. Kepczyńska E. (2017). Identification of LEC1, L1L and polycomb repressive complex 2 genes and their expression during the induction phase of *Medicago truncatula* Gaertn. somatic embryogenesis. Plant Cell Tissue Organ Cult. 129, 119–132. 10.1007/s11240-016-1161-8

[B85] Palatnik J. F. Allen E. Wu X. Schommer C. Schwab R. Carrington J. C. . (2003). Control of leaf morphogenesis by microRNAs. Nature 425, 257–263. 10.1038/nature01958 12931144

[B86] Palovaara J. De Zeeuw T. Weijers D. (2016). Tissue and organ initiation in the plant embryo: a first time for everything. Ann. Rev. Cell Dev. Biol. 32, 47–75. 10.1146/annurev-cellbio-111315-124929 27576120

[B87] Paul P. Joshi S. Tian R. Junior R. D. Chakrabarti M. Perry S. E. (2021). The MADS-domain factor AGAMOUS-Like18 promotes somatic embryogenesis. Plant Physiol. 188, 1617–1631. 10.1093/plphys/kiab553 34850203 PMC8896631

[B88] Pelletier J. M. Kwong R. W. Park S. Le B. H. Baden R. Cagliaria A. . (2017). LEC1 sequentially regulates the transcription of genes involved in diverse developmental processes during seed development. Proc. Natl. Acad. Sci. U.S.A. 114, E6710–E6719. 10.1073/pnas.1707957114 28739919 PMC5559047

[B89] Perry S. E. Lehti M. D. Fernandez D. E. (1999). The MADS-domain protein AGAMOUS-like 15 accumulates in embryonic tissues with diverse origins. Plant Physiol. 120, 121–129. 10.1104/pp.120.1.121 10318690 PMC59244

[B90] Perry S. E. Nichols K. W. Fernandez D. E. (1996). The MADS domain protein AGL15 localizes to the nucleus during early stages of seed development. Plant Cell 8, 1977–1989. 10.1105/tpc.8.11.1977 8953767 PMC161328

[B91] Piya S. Shrestha S. K. Binder B. Stewart C. N. Jr. Hewezi T. (2014). Protein-protein interaction and gene co-expression maps of ARFs and Aux/IAAs in Arabidopsis. Front. Plant Sci. 5, 744. 10.3389/fpls.2014.00744 PMC4274898 25566309

[B92] Plotnikova A. Kellner M. J. Schon M. A. Mosiolek M. Nodine M. D. (2019). MicroRNA dynamics and functions during Arabidopsis embryogenesis. Plant Cell 31, 2929–2946. 10.1105/tpc.19.00395 31562217 PMC6925019

[B93] Quiroz-Figueroa F. R. Rojas-Herrera R. Galaz-Avalos R. M. Loyola-Vargas V. M. (2006). Embryo production through somatic embryogenesis can be used to study cell differentiation in plants. Plant Cell Tissue Organ Cult. 86, 285. 10.1007/s11240-006-9139-6

[B94] Raghavan C. Ong E. K. Dalling M. J. Stevenson T. W. (2006). Regulation of genes associated with auxin, ethylene and ABA pathways by 2, 4-dichlorophenoxyacetic acid in Arabidopsis. Funct. Integrat. Genom. 6, 60–70. 10.1007/s10142-005-0012-1 16317577

[B95] Rhoades M. W. Reinhart B. J. Lim L. P. Burge C. B. Bartel B. Bartel D. P. (2002). Prediction of plant microRNA targets. Cell 110, 513–520. 10.1016/S0092-8674(02)00863-2 12202040

[B96] Rodríguez-Sanz H. Moreno-Romero J. Solís M.-T. Köhler C. Risueño M. C. Testillano P. S. (2014). Changes in histone methylation and acetylation during microspore reprogramming to embryogenesis occur concomitantly with BnHKMT and BnHAT expression and are associated with cell totipotency, proliferation, and differentiation in Brassica napus. Cytogenet. Genome Res. 143, 209–218. 10.1159/000365261 25060767

[B97] Rose R. J (2019). Somatic embryogenesis in the *Medicago truncatula* model: cellular and molecular mechanisms. Front. Plant Sci. 10, 267. 10.3389/fpls.2019.00267 PMC6447896 30984208

[B98] Ruan J. Chen H. Zhu T. Yu Y. Lei Y. Yuan L. . (2021). Brassinosteroids repress the seed maturation program during the seed-to-seedling transition. Plant Physiol. 186, 534–548. 10.1093/plphys/kiab089 33620498 PMC8154094

[B99] Rubio-Somoza I. Weigel D. (2011). MicroRNA networks and developmental plasticity in plants. Trends Plant Sci. 16, 258–264. 10.1016/j.tplants.2011.03.001 21466971

[B100] Salvo S. A. Hirsch C. N. Buell C. R. Kaeppler S. M. Kaeppler H. F. (2014). Whole transcriptome profiling of maize during early somatic embryogenesis reveals altered expression of stress factors and embryogenesis-related genes. PLoS ONE 9, e111407. 10.1371/journal.pone.0111407 25356773 PMC4214754

[B101] Saptari R. T. Susila H. (2019). Data mining study of hormone biosynthesis gene expression reveals new aspects of somatic embryogenesis regulation. In Vitro Cellular Dev. Biol.-Plant 55, 139–152. 10.1007/s11627-018-9947-5

[B102] Sato A. Yamamoto K. T. (2008). Overexpression of the non-canonical Aux/IAA genes causes auxin-related aberrant phenotypes in Arabidopsis. Physiol. Plant. 133, 397–405. 10.1111/j.1399-3054.2008.01055.x 18298415

[B103] Sharp W (1980). The physiology of *in vitro* asexual embryogenesis. Horticultural Rev. 2, 268–310. 10.1002/9781118060759.ch6

[B104] Song X. Li Y. Cao X. Qi Y. (2019). MicroRNAs and their regulatory roles in plant–environment interactions. Ann. Rev.Plant Biol. 70, 489–525. 10.1146/annurev-arplant-050718-100334 30848930

[B105] Stepanova A. N. Robertson-Hoyt J. Yun J. Benavente L. M. Xie D.-Y. DoleŽal K. . (2008). TAA1-mediated auxin biosynthesis is essential for hormone crosstalk and plant development. Cell 133, 177–191. 10.1016/j.cell.2008.01.047 18394997

[B106] Steunou A.-L. Rossetto D. Côté J. (2014). “Regulating chromatin by histone acetylation,” in Fundamentals of Chromatin, eds J. Workman and S. Abmayr (New York, NY: Springer), 147–212. 10.1007/978-1-4614-8624-4_4

[B107] Stricker S. H. Köferle A. Beck S. (2017). From profiles to function in epigenomics. Nat. Rev. Genet. 18, 51–66. 10.1038/nrg.2016.138 27867193

[B108] Su Y. H. Tang L. P. Zhao X. Y. Zhang X. S. (2021). Plant cell totipotency: Insights into cellular reprogramming. J. Integrative Plant Biol. 63, 228–243. 10.1111/jipb.12972 32437079

[B109] Sugimoto K. Jiao Y. L. Meyerowitz E. M. (2010). Arabidopsis regeneration from multiple tissues occurs via a root development pathway. Dev. Cell 18, 463–471. 10.1016/j.devcel.2010.02.004 20230752

[B110] Suzuki M. Wang H. H.-Y. Mccarty D. R. (2007). Repression of the LEAFY COTYLEDON 1/B3 regulatory network in plant embryo development by VP1/ABSCISIC ACID INSENSITIVE 3-LIKE B3 genes. Plant Physiol. 143, 902–911. 10.1104/pp.106.092320 17158584 PMC1803726

[B111] Szyrajew K. Bielewicz D. Dolata J. Wójcik A. M. Nowak K. Szczygiel-Sommer A. . (2017). MicroRNAs are intensively regulated during induction of somatic embryogenesis in Arabidopsis. Front. Plant Sci. 8, 18. 10.3389/fpls.2017.00018 28167951 PMC5253390

[B112] Takanashi H. Sumiyoshi H. Mogi M. Hayashi Y. Ohnishi T. Tsutsumi N. (2018). miRNAs control HAM1 functions at the single-cell-layer level and are essential for normal embryogenesis in Arabidopsis. Plant Mol. Biol. 96, 627–640. 10.1007/s11103-018-0719-8 29574557

[B113] Tang G. Reinhart B. J. Bartel D. P. Zamore P. D. (2003). A biochemical framework for RNA silencing in plants. Genes Dev. 17, 49–63. 10.1101/gad.1048103 12514099 PMC195971

[B114] Tang W. Perry S. E. (2003). Binding site selection for the plant MADS Domain protein AGL15: an *in vitro* and *in vivo* study. J. Biol. Chem. 278, 28154–28159. 10.1074/jbc.M212976200 12743119

[B115] Thakare D. Tang W. Hill K. Perry S. E. (2008). The MADS-domain transcriptional regulator AGAMOUS-LIKE15 promotes somatic embryo development in Arabidopsis and soybean. Plant Physiol. 146, 1663–1672. 10.1104/pp.108.115832 18305206 PMC2287341

[B116] Thorpe T. A. Stasolla C. (2001). “Somatic embryogenesis,” in Current trends in the Embryology of Angiosperms, eds S. S. Bhojwani and W. Y. Soh (Dordrecht: Springer), 279–336. 10.1007/978-94-017-1203-3_12

[B117] Tian R. Paul P. Joshi S. Perry S. E. (2020a). Genetic activity during early plant embryogenesis. Biochemical J. 477, 3743–3767. 10.1042/BCJ20190161 33045058 PMC7557148

[B118] Tian R. Wang F. Zheng Q. Niza V. M. A. G. E. Downie A. B. (2020b). Direct and indirect targets of the Arabidopsis seed transcription factor ABSCISIC ACID INSENSITIVE3. Plant J. 103, 1679–1694. 10.1111/tpj.14854 32445409

[B119] Toonen M. A. J. Schmidt E. D. L. Vankammen A. Devries S. C. (1997). Promotive and inhibitory effects of diverse arabinogalactan proteins on *Daucus carota* L. somatic embryogenesis. Planta 203, 188–195. 10.1007/s004250050181

[B120] Tvorogova V. E. Fedorova Y. A. Potsenkovskaya E. A. Kudriashov A. A. Efremova E. P. Kvitkovskaya V. A. . (2019). The WUSCHEL-related homeobox transcription factor MtWOX9-1 stimulates somatic embryogenesis in *Medicago truncatula*. Plant Cell Tissue Organ Cult. 138, 517–527. 10.1007/s11240-019-01648-w

[B121] Van Hengel A. J. Guzzo F. Van Kammen A. De Vries S. C. (1998). Expression pattern of the carrot EP3 endochitinase genes in suspension cultures and in developing seeds. Plant Physiol. 117, 43–53. 10.1104/pp.117.1.43 9576773 PMC35020

[B122] Van Hengel A. J. Tadesse Z. Immerzeel P. Schols H. Van Kammen A. De Vries S. C. (2001). N-acetylglucosamine and glucosamine-containing arabinogalactan proteins control somatic embryogenesis. Plant Physiol. 125, 1880–1890. 10.1104/pp.125.4.1880 11299367 PMC88843

[B123] Van Hengel A. J. Van Kammen A. De Vries S. C. (2002). A relationship between seed development, Arabinogalactan-proteins (AGPs) and the AGP mediated promotion of somatic embryogenesis. Physiol. Plant. 114, 637–644. 10.1034/j.1399-3054.2002.1140418.x 11975739

[B124] Vandenbussche F. Vaseva I. Vissenberg K. Van Der Straeten D. (2012). Ethylene in vegetative development: a tale with a riddle. New Phytol. 194, 895–909. 10.1111/j.1469-8137.2012.04100.x 22404712

[B125] Veerappan V. Chen N. Reichert A. I. Allen R. D. (2014). HSI2/VAL1 PHD-like domain promotes H3K27 trimethylation to repress the expression of seed maturation genes and complex transgenes in Arabidopsis seedlings. BMC Plant Biol. 14, 1–19. 10.1186/s12870-014-0293-4 25367506 PMC4232687

[B126] Von Arnold S. Sabala I. Bozhkov P. Dyachok J. Filonova L. (2002). Developmental pathways of somatic embryogenesis. Plant Cell Tissue Organ Cult. 69, 233–249. 10.1023/A:1015673200621

[B127] Wang F. Perry S. E. (2013). Identification of direct targets of FUSCA3, a key regulator of Arabidopsis seed development. Plant Physiol. 161, 1251–1264. 10.1104/pp.112.212282 23314941 PMC3585594

[B128] Wang F. X. Shang G. D. Wu L. Y. Xu Z. G. Zhao X. Y. Wang J. W. (2020). Chromatin accessibility dynamics and a hierarchical transcriptional regulatory network structure for plant somatic embryogenesis. Dev. Cell 54, 742–757. 10.1016/j.devcel.2020.07.003 32755547

[B129] Wang H. Caruso L. V. Downie A. B. Perry S. E. (2004). The embryo MADS domain protein AGAMOUS-Like 15 directly regulates expression of a gene encoding an enzyme involved in gibberellin metabolism. Plant Cell 16, 1206–1219. 10.1105/tpc.021261 15084721 PMC423210

[B130] Weiss D. Ori N. (2007). Mechanisms of cross talk between gibberellin and other hormones. Plant Physiol. 144, 1240–1246. 10.1104/pp.107.100370 17616507 PMC1914132

[B131] Willmann M. R. Mehalick A. J. Packer R. L. Jenik P. D. (2011). MicroRNAs regulate the timing of embryo maturation in Arabidopsis. Plant Physiol. 155, 1871–1884. 10.1104/pp.110.171355 21330492 PMC3091098

[B132] Wójcik A. M. Gaj M. D. (2016). miR393 contributes to the embryogenic transition induced *in vitro* in Arabidopsis via the modification of the tissue sensitivity to auxin treatment. Planta 244, 231–243. 10.1007/s00425-016-2505-7 27040841 PMC4903112

[B133] Wójcik A. M. Wójcikowska B. Gaj M. D. (2020). Current perspectives on the auxin-mediated genetic network that controls the induction of somatic embryogenesis in plants. Int. J. Mol. Sci. 21, 1333. 10.3390/ijms21041333 32079138 PMC7072907

[B134] Wójcikowska B. Gaj M. D. (2016). “Somatic embryogenesis in arabidopsis,” in Somatic Embryogenesis: Fundamental Aspects and Applications, eds V. Loyola-Vargas and N. Ochoa-Alejo (Cham: Springer), 185–199. 10.1007/978-3-319-33705-0_11

[B135] Wójcikowska B. Gaj M. D. (2017). Expression profiling of AUXIN RESPONSE FACTOR genes during somatic embryogenesis induction in Arabidopsis. Plant Cell Rep. 36, 843–858. 10.1007/s00299-017-2114-3 28255787 PMC5486788

[B136] Wójcikowska B. Jaskola K. Gasiorek P. Meus M. Nowak K. Gaj M. D. (2013). LEAFY COTYLEDON2 (LEC2) promotes embryogenic induction in somatic tissues of Arabidopsis, via YUCCA-mediated auxin biosynthesis. Planta 238, 425–440. 10.1007/s00425-013-1892-2 23722561 PMC3751287

[B137] Wójcikowska B. Wójcik A. M. Gaj M. D. (2020). Epigenetic regulation of auxin-induced somatic embryogenesis in plants. Int. J. Mol. Sci. 21, 2307. 10.3390/ijms21072307 32225116 PMC7177879

[B138] Xu K. D. Liu K. Wu J. X. Wang W. Zhu Y. L. Li C. J. . (2018). A MADS-box gene associated with protocorm-like body formation in Rosa canina alters floral organ development in Arabidopsis. *Canad. J*. Plant Sci. 98, 309–317. 10.1139/cjps-2017-0027

[B139] Yang X. Zhang X. (2010). Regulation of somatic embryogenesis in higher plants. Crit. Rev. Plant Sci. 29, 36–57. 10.1080/07352680903436291

[B140] Yang Z. Li C. Wang Y. Zhang C. Wu Z. Zhang X. . (2014). GhAGL15s, preferentially expressed during somatic embryogenesis, promote embryogenic callus formation in cotton (*Gossypium hirsutum* L.). Mol. Genet. Genomics 289, 873–883. 10.1007/s00438-014-0856-y 24833045

[B141] Yu Y. Jia T. Chen X. (2017). The 'how' and 'where' of plant microRNAs. New Phytol. 216, 1002–1017. 10.1111/nph.14834 29048752 PMC6040672

[B142] Zheng Q. Perry S. E. (2014). Alterations in the transcriptome of soybean in response to enhanced somatic embryogenesis promoted by orthologs of AGAMOUS-Like15 and AGAMOUS-Like18. Plant Physiol. 164, 1365–1377. 10.1104/pp.113.234062 24481137 PMC3938626

[B143] Zheng Q. Zheng Y. Ji H. Burnie W. Perry S. E. (2016). Gene regulation by the AGL15 transcription factor reveals hormone interactions in somatic embryogenesis. Plant Physiol. 172, 2374–2387. 10.1104/pp.16.00564 27794101 PMC5129705

[B144] Zheng Q. Zheng Y. Perry S. E. (2013a). AGAMOUS-Like15 promotes somatic embryogenesis in Arabidopsis and soybean in part by the control of ethylene biosynthesis and response. Plant Physiol. 161, 2113–2127. 10.1104/pp.113.216275 23457229 PMC3613480

[B145] Zheng Q. Zheng Y. Perry S. E. (2013b). Decreased GmAGL15 expression and reduced ethylene synthesis may contribute to reduced somatic embryogenesis in a poorly embryogenic cultivar of Glycine max. Plant Signal. Behav. 8, e25422. 10.4161/psb.25422 23838957 PMC4002625

[B146] Zheng Y. Ren N. Wang H. Stromberg A. J. Perry S. E. (2009). Global identification of targets of the Arabidopsis MADS domain protein AGAMOUS-Like15. Plant Cell 21, 2563–2577. 10.1105/tpc.109.068890 19767455 PMC2768919

[B147] Zhu C. Perry S. E. (2005). Control of expression and autoregulation of AGL15, a member of the MADS-box family. Plant J. 41, 583–594. 10.1111/j.1365-313X.2004.02320.x 15686521

[B148] Zimmerman J. L (1993). Somatic embryogenesis: a model for early development in higher plants. Plant Cell 5, 1411–1423. 10.2307/3869792 12271037 PMC160372

