# Supplementary material for: AGL15 Promotion of Somatic Embryogenesis: Role and Molecular Mechanism
Source: Front Plant Sci. 2022 Mar 28;13:861556. doi: 10.3389/fpls.2022.861556 (PMC8996056; doi:10.3389/fpls.2022.861556)
Supplement: Supplementary file 3 [file Data_Sheet_1.PDF]

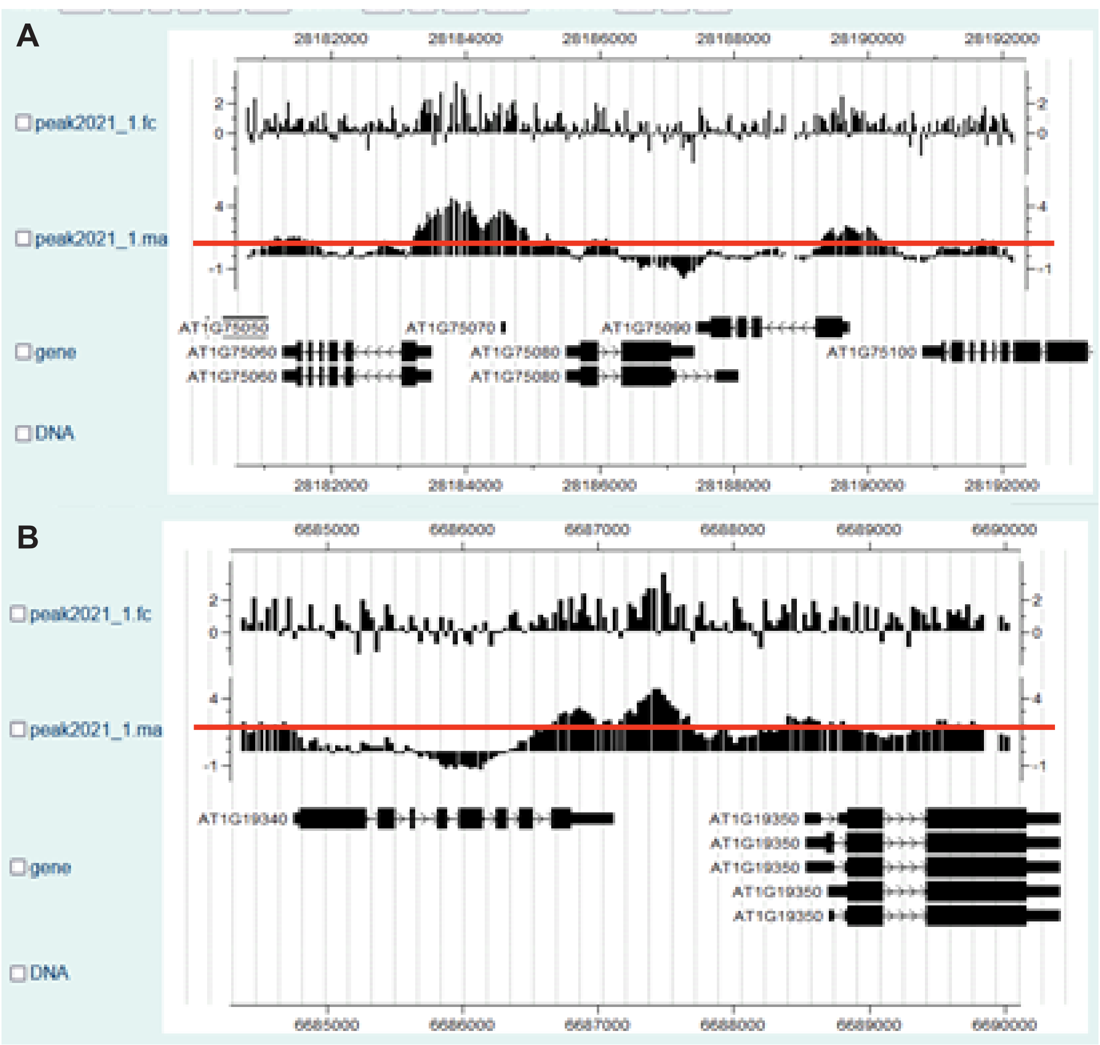

Supplementary Figure S1. AGL15 association with:
(A) *BZR1* (At1g75080) and
(B) *BES1* (At1g19350)
as assessed from ChIP-chip experiments (Zheng et al., 2009) using CisGenome (Ji et al., 2008). The red line indicates a two-fold enrichment of co-precipitation of the fragment compared to control.
